# The diagnostic performance of functional dopaminergic scintigraphic imaging in the diagnosis of dementia with Lewy bodies: an updated systematic review

**DOI:** 10.1007/s00259-023-06154-y

**Published:** 2023-03-15

**Authors:** Mario Jreige, George K. Kurian, Jérémy Perriraz, Jevita Potheegadoo, Fosco Bernasconi, Sara Stampacchia, Olaf Blanke, Griffa Alessandra, Noemie Lejay, Paolo Salvioni Chiabotti, Olivier Rouaud, Marie Nicod Lalonde, Niklaus Schaefer, Giorgio Treglia, Gilles Allali, John O. Prior

**Affiliations:** 1 Department of Nuclear Medicine and Molecular Imaging, Lausanne University Hospital, Rue du Bugnon 46, CH-1011 Lausanne, Switzerland; 2 Faculty of Biology and Medicine, University of Lausanne, Lausanne, Switzerland; 3 Laboratory of Cognitive Neuroscience, Neuro-X Institute & Brain Mind Institute, Faculty of Life Sciences, Swiss Federal Institute of Technology (EPFL), Geneva, Switzerland; 4 Leenaards Memory Center, Department of Clinical Neurosciences, Lausanne University Hospital, Lausanne, Switzerland; 5 Clinic of Nuclear Medicine, Imaging Institute of Southern Switzerland, Ente Ospedaliero Cantonale, 6500 Bellinzona, Switzerland; 6 Faculty of Biomedical Sciences, Università Della Svizzera Italiana, 6900 Lugano, Switzerland

**Keywords:** Dopaminergic imaging, DATscan, 123-FP-CIT, Dementia with Lewy bodies, DLB

## Abstract

**Introduction:**

Dopaminergic scintigraphic imaging is a cornerstone to support the diagnosis in dementia with Lewy bodies. To clarify the current state of knowledge on this imaging modality and its impact on clinical diagnosis, we performed an updated systematic review of the literature.

**Methods:**

This systematic review was carried out according to PRISMA guidelines. A comprehensive computer literature search of PubMed/MEDLINE, EMBASE, and Cochrane Library databases for studies published through June 2022 was performed using the following search algorithm: (a) "Lewy body" [TI] OR "Lewy bodies" [TI] and (b) ("DaTscan" OR "ioflupane" OR "123ip" OR "123?ip" OR "123 ip" OR "123i-FP-CIT" OR "FPCIT" OR "FP-CIT" OR "beta?CIT" OR "beta CIT" OR "CIT?SPECT" OR "CIT SPECT" OR "Dat?scan*" OR "dat scan*" OR "dat?spect*" OR "SPECT"). Risk of bias and applicability concerns of the studies were evaluated using the QUADAS-2 tool.

**Results:**

We performed a qualitative analysis of 59 studies. Of the 59 studies, 19 (32%) addressed the diagnostic performance of dopamine transporter imaging, 15 (25%) assessed the identification of dementia with Lewy bodies in the spectrum of Lewy body disease and 18 (31%) investigated the role of functional dopaminergic imaging in distinguishing dementia with Lewy bodies from other dementias. Dopamine transporter loss was correlated with clinical outcomes in 19 studies (32%) and with other functional imaging modalities in 15 studies (25%). Heterogeneous technical aspects were found among the studies through the use of various radioligands, the more prevalent being the [123I]N‑ω‑fluoropropyl‑2β‑carbomethoxy‑3β‑(4‑iodophenyl) nortropane (^123^I-FP-CIT) in 54 studies (91.5%). Image analysis used visual analysis (9 studies, 15%), semi-quantitative analysis (29 studies, 49%), or a combination of both (16 studies, 27%).

**Conclusion:**

Our systematic review confirms the major role of dopaminergic scintigraphic imaging in the assessment of dementia with Lewy bodies. Early diagnosis could be facilitated by identifying the prodromes of dementia with Lewy bodies using dopaminergic scintigraphic imaging coupled with emphasis on clinical neuropsychiatric symptoms. Most published studies use a semi-quantitative analytical assessment of tracer uptake, while there are no studies using quantitative analytical methods to measure dopamine transporter loss. The superiority of a purely quantitative approach to assess dopaminergic transmission more accurately needs to be further clarified.

**Supplementary Information:**

The online version contains supplementary material available at 10.1007/s00259-023-06154-y.

## Introduction

Dementia with Lewy bodies (DLB) is the second most frequent type of neurodegenerative dementia after Alzheimer’s disease (AD), comprising 15–25% of all dementias [1]. Neuropathological findings in patients with DLB show Lewy bodies and Lewy neurites that are positive for α-synuclein immunohistochemical staining, as well as neuronal degeneration in the neocortex, limbic system and brainstem [2]. Although a clear-cut distinction between the entities of the “Lewy body disease (LBD) spectrum” (dementia with Lewy bodies, idiopathic Parkinson’s disease (PD) and PD with dementia) is not always easy, diagnosis of DLB is made clinically through the identification of core clinical features. These include fluctuations of attention and cognitive impairment, visual hallucinations, rapid eye-movement (REM) sleep behavior disorder (RBD), and parkinsonism. Reduced dopamine transporter uptake in the basal ganglia shown by single-positron emission computed tomography (SPECT) is included as an indicative biomarker in the fourth and latest consensus on the diagnosis of Lewy body dementia [3]. Dopamine transporter (DAT) imaging is performed using specific radioligands. One such ligand is [^123^I]N‑ω‑fluoropropyl‑2β‑carbomethoxy‑3β‑(4‑iodophenyl) nortropane (^123^I-FP-CIT), a cocaine analogue that specifically binds to presynaptic DATs in the central nervous system, thus identifying the location and concentration of dopamine transporters in the synapses of dopamine-secreting neurons of the corpus striatum in the central nervous system. This allows imaging of the nigrostriatal pathway denervation that occurs in DLB. Other radiotracers can also be used [4]. Clinically, DLB can be difficult to differentiate from other forms of dementia. Furthermore, it is of paramount clinical importance to differentiate DLB from other etiologies as the subsequent clinical and therapeutical management of patients varies, especially to avoid any inappropriate use of neuroleptics in DLB patients [5]. The course of the disease is also different, as life expectancy is shorter in DLB. Literature covering these topics lack homogeneity.

A systematic review and a Bayesian latent class model (LCM) meta-analysis on the diagnostic accuracy of both DAT SPECT imaging as well as metaiodobenzylguanidine (MIBG) myocardial scintigraphy in DLB diagnosis has been published previously [6]. However, there were several limitations, as the literature was only reviewed up to the year 2018 and the number of analyzed studies was small (*n* = 27), out of which less than a third used the new criteria of DLB published in 2017 [3]. In the present systematic literature review, we present an updated analysis of dopaminergic transporter imaging in the diagnosis of DLB.

## Methods

This study was carried out according to the Preferred Reporting Items for Systematic Reviews and Meta-Analyses (PRISMA) guidelines, which describe an evidence-based minimum set of items for reporting in systematic reviews and meta-analyses [7]. A predefined protocol was created by the authors (without registration).

### Search strategy

Two authors (MJ and GKK) performed a comprehensive computer literature search of the PubMed/MEDLINE, EMBASE and Cochrane Library databases to identify relevant retrospective or prospective published studies on the diagnostic performance of functional dopaminergic scintigraphy in the diagnosis of Lewy body dementia. The search algorithm used was based on a combination of terms, as follows: (a) "Lewy body" [TI] OR "Lewy bodies" [TI] and (b) ("DaTscan" OR "ioflupane" OR "123ip" OR "123?ip" OR "123 ip" OR "123i-FP-CIT" OR "FPCIT" OR "FP-CIT" OR "beta?CIT" OR "beta CIT" OR "CIT?SPECT" OR "CIT SPECT" OR "Dat?scan*" OR "dat scan*" OR "dat?spect*" OR "SPECT"). The search was updated through June 2022. No language restriction was applied. To expand the search, references of the retrieved articles were also screened for additional studies.

### Study selection

Studies or subsets of studies investigating the diagnostic performance of functional dopaminergic scintigraphy in the evaluation of patients with Dementia with Lewy bodies (DLB) were eligible for *inclusion* in the qualitative analysis (systematic review).

The *exclusion criteria* were as follows: (a) articles not within the field of interest of this review, such as those with outcomes unrelated to dopaminergic scintigraphic imaging for diagnosis of DLB (e.g., use of brain perfusion SPECT or PET or myocardial scintigraphy *alone*); (b) review articles, editorials or letters, comments, conference proceedings; (c) case reports or small case series (< 5 patients).

Two researchers (MJ and GKK) independently reviewed the titles and abstracts of the retrieved articles, applying the inclusion and exclusion criteria mentioned above. Articles were rejected if they were clearly ineligible. The same two researchers then independently reviewed the full-text versions of the remaining articles to determine their eligibility for inclusion. Disagreements were resolved in a consensus meeting.

### Data extraction

Two researchers independently performed the data extraction. For each potentially eligible study, information was collected concerning basic study characteristics (authors, year of publication, country of origin, study design), patient characteristics (type and number of patients, mean age, sex ratio) and technical aspects (radiotracer used, hybrid imaging modality, mean injected activity, time interval between radiotracer injection and image acquisition, image analysis). Finally, information about the main outcome of this systematic review (diagnostic performance of dopaminergic scintigraphic imaging) was collected. Diagnostic performance was assessed according to clinical confirmation of DLB diagnosis as well as post-mortem neuropathological studies, the latter being rarely systematically documented. Differences between basic study characteristics, technical aspects and outcomes were reported and were analyzed.

### Quality assessment

**Fig. 1:**
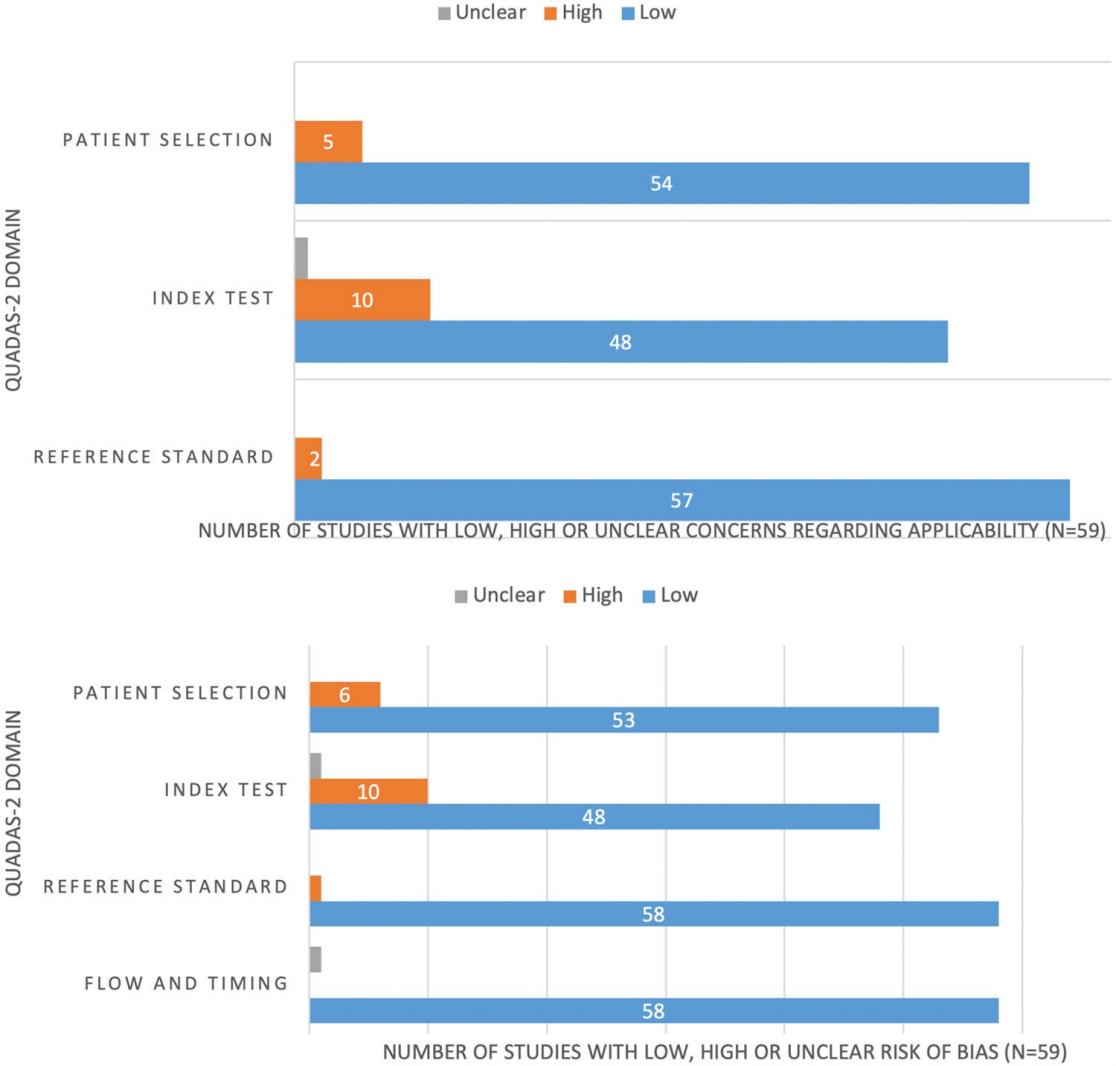
QUADAS-2 graphical results

The overall quality of the studies included in the systematic review was critically appraised based on the revised Quality Assessment of Diagnostic Accuracy Studies tool (QUADAS-2) [8]. This tool comprises four domains: patient selection, index test, reference standard, and flow and timing. Three independent reviewers (MJ, GKK, JP) assessed each domain in terms of risk of bias (i.e., selection bias, as well as biases concerning the index test, reference standard and timing of studies), and the first three domains were also assessed in terms of concerns regarding applicability [8] (Table 1 and Fig. 1).

**Table 1:** Quality assessment of the studies included in the systematic review according to the QUADAS-2 tool [8]

Authors	Patient selection		Index test		Reference standard		Flow and timing
	Risk of bias (unclear, Low, High)	Applicability concerns	Risk of bias	Applicability concerns	Risk of bias	Applicability concerns	Risk of bias
Ceravolo et al. [49]	Low	Low	Low	Low	Low	Low	low
Chen et al. [15]	Low	Low	Low	Low	Low	Low	Low
Chiu et al. [60]	Low	Low	High	High	Low	Low	Low
Colloby et al. [25]	Low	Low	High	High	Low	Low	Low
Colloby et al. [27]	Low	Low	Low	Low	Low	Low	Low
Colloby et al. [28]	Low	Low	Low	Low	Low	Low	Low
Del Sole et al. [61]	Low	Low	Low	Low	Low	Low	Low
Donaghy et al. [62]	Low	Low	High	High	Low	Low	Low
Durcan et al. [63]	Low	Low	High	High	Low	Low	Low
Gupta et al. [9]	Low	Low	Low	Low	Low	Low	Low
Hansen et al. [64]	Low	Low	Low	Low	Low	Low	Low
Huber et al. [10]	Low	Low	Low	Low	Low	Low	Low
Iizuka et al. [11]	Low	Low	Low	Low	Low	Low	Low
Inagawa et al. [16]	Low	Low	Low	Low	Low	Low	Low
Iwabuchi et al. [26]	Low	Low	Low	Low	Low	Low	Low
Joling et al. [29]	High	High	Low	Low	Low	Low	Low
Joling et al. [30]	Low	Low	Unclear	Unclear	Low	Low	Low
Kamagata et al. [31]	Low	Low	Unclear	Unclear	Low	Low	Low
Kasanuki et al. [32]	Low	Low	Low	Low	Low	Low	Low
Kemp et al. [50]	Low	Low	High	High	Low	Low	Low
Kobayashi et al. [17]	Low	Low	Low	Low	Low	Low	Low
Lamotte et al. [33]	Low	Low	Low	Low	Low	Low	Low
Lim et al. [12]	Low	Low	High	High	Low	Low	Low
Lloyd et al. [51]	Low	Low	High	High	Low	Low	Low
Maltais et al. [52]	Low	Low	Low	Low	Low	Low	Low
McKeith et al. [68]	Low	Low	Low	Low	Low	Low	Low
Miyagawa et al. [13]	Low	Low	Low	Low	Low	Low	Low
Miyamoto et al. [58]	Low	Low	Low	Low	Low	Low	Low
Morgan et al. [34]	High	High	High	High	Low	Low	Low
Nakahara et al. [18]	Low	Low	Low	Low	Low	Low	Low
Nicastro et al. [35]	Low	Low	Low	Low	Low	Low	Low
Nicastro et al. [53]	Low	Low	Low	Low	Low	Low	Low
Nicastro et al. [14]	Low	Low	Low	Low	Low	Low	Low
O’Brien et al. [36]	Low	Low	Low	Low	Low	Low	Low
O’Brien et al. [37]	Low	Low	Low	Low	Low	Low	Low
Oliveira et al. [59]	Low	Low	Low	Low	Low	Low	Low
Pilotto et al. [38]	Low	Low	Low	Low	Low	Low	Low
Ransmayr et al. [39]	High	High	Low	Low	Low	Low	Unclear
Roberts et al. [19]	Low	Low	Low	Low	Low	Low	Low
Roberts et al. [54]	Low	Low	Low	Low	Low	Low	Low
Roselli et al. [40]	High	Low	Low	Low	Low	Low	Low
Sakamoto et al. [20]	High	Low	Low	Low	Low	Low	Low
Shimizu et al. [21]	Low	Low	Low	Low	Low	Low	Low
Shimizu et al. [22]	Low	Low	Low	Low	Low	Low	Low
Siepel et al. [65]	Low	Low	Low	Low	Low	Low	Low
Siepel et al. [41]	Low	Low	High	High	Low	Low	Low
Spehl et al. [42]	High	High	Low	Low	Low	Low	Low
Taylor et al. [43]	Low	Low	Low	Low	Low	Low	Low
Thomas et al. [55]	Low	Low	Low	Low	Low	Low	Low
Tiraboschi et al. [23]	Low	Low	Low	Low	Low	Low	Low
Treglia et al. [24]	Low	Low	Low	Low	Low	High	Low
Van de Beek et al. [44]	Low	Low	Low	Low	Low	Low	Low
Van der Zande et al. [45]	Low	Low	Low	Low	Low	Low	Low
Van der Zande et al. [46]	Low	Low	Low	Low	Low	Low	Low
Walker et al. [66]	Low	Low	Low	Low	Low	Low	Low
Walker et al. [47]	Low	Low	Low	Low	High	High	Low
Walker et al. [56]	Low	Low	Low	Low	Low	Low	Low
Walker et al. [57]	Low	High	High	High	Low	Low	Low
Ziebell et al. [48]	Low	Low	Low	Low	Low	Low	Low

## Results

### Literature search

**Fig. 2:**
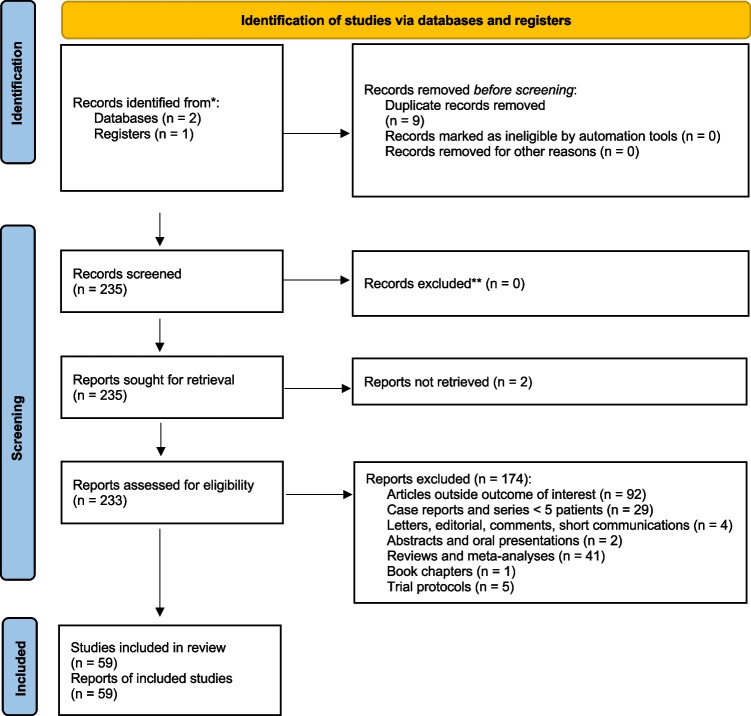
Flow chart of the search for eligible studies on the diagnostic performance of radiolabelled ^123^I-FP-CIT SPECT in detecting dopaminergic denervation in patients with Lewy body dementia

A comprehensive computer literature search of the PubMed/MEDLINE, EMBASE and Cochrane Library databases revealed 218 peer-reviewed articles. Upon review of titles and abstracts, 153 articles were excluded, as follows: 79 were not in the field of interest of this review, 44 were reviews, editorials or letters, and 30 were case reports or small case series (< 5 patients). 59 were selected and retrieved in full-text version [3–61]. No additional studies were found by screening the references of these articles (Fig. 2).

Finally, 59 articles including data on the diagnostic performance of functional dopaminergic scintigraphic imaging in the diagnosis of dementia with Lewy bodies (DLB) dementia were eligible for the qualitative analysis (systematic review) [3–61]. The characteristics of the studies included in the systematic review are summarized in Tables 2 and 3.

**Table 2:** Methodology and outcome summary of the included studies

Authors	Study objectives	Variables analyzed		Statistical analysis	Outcome summary
		SPECT variables	Clinical variables		
Ceravolo et al. [49]	Assess dopamine transporter function	Ratio of specific (bilateral caudate nucleus, putamen) to non-specific (occipital cortex) radiotracer binding	Age, sex, MMSE, CAMCOG, NPI, UPDRS	NR	Decreased specific to non-specific FP-CIT binding in DLB compared to AD
Chen et al. [15]	Determine clinical phenotypes associated with beta-amyloid PET (A+) and DaT imaging (D+) in MCI-LB (higher risk of progression to probable DLB)	Putamen z score (cutoff <-0.82), beta-amyloid PET PiB SUVR	MMSE, CDR sum of boxes and UPDRS-III scores, visual hallucinations, fluctuations, parkinsonism, probable RBD	T test for continuous variables, Chi-squared test for categorical variables; linear or logistic regression models for predictors (factorial modeling approach using continuous A and D); Age adjusted.	Decreased putaminal z score and lower PiB SUVRs independently associated with higher UPDRS-III scores; More probability of RBD in positive DaTscan group
Chiu et al. [60]	Differentiate DLB from AD with motor dysfunction	SBR and visual rating	Novel designed questionnaire, composite scale with motor dysfunction questionnaire (MDQ) and dopamine transporter imaging	Chi-square test for each question in the HAI-MDQ between DLB and non-DLB groups,	Higher frequency of motor dysfunction in DLB group; Lower SBR in DLB group; Sensitivity/Specificity (%): 91/72 (MDQ), 91/80 (SBR), 87/93 (composite scale)
Colloby et al. [25]	Compare diagnostic accuracy of ^99m^Tc-exametazime to ^123^I-FP-CIT for AD and DLB	Normal or Abnormal visual rating of 123I-FP-CIT SPECT Scans (grade 0 = normal, grade 1,2,3 = abnormal).	Age, sex, MMSE, CAMCOG, UPDRS-III, duration of illness	Shapiro-Wilk test for normality of distribution of continuous variables; one-way ANOVA (F-Test) for group effects; Nominal data with Chi-square tests; Cohen’s kappa test for inter-observer agreement between raters’ visual assessment; ROC curve analysis for Sn and Sp	Excellent Inter-rater agreement (κ = 0.88) for DaTscan; Superior ROC for DaTscan in diagnosing DLB (AUC 0.83, sensitivity: 78.6%, specificity: 87.9) compared to brain perfusion
Colloby et al. [27]	Investigate rate of progression of nigrostriatal dopaminergic loss in DLB, PD, PDD using DaTscan	Striatal binding between groups	Age, MMSE, UPDRS III, duration of symptoms	Shapiro-Wilk test for normality of distribution of continuous variables; Pearson’s r or Spearman’s rho as appropriate for correlations between SPECT and clinical variables; 2-sample dependent t tests for differences in binding rations; ANCOVA (analysis of covariance) for rates of decline in BRs	Faster rates of decline in striatal binding in LBD than controls in caudate nucleus and posterior putamen; Faster rates of decline in striatal binding in DLB & PDD than controls in anterior putamen; Similar rates of decline between DLB, PD and PDD
Colloby et al. [28]	Investigate the differences in striatal binding using automated statistical parametric mapping (SPM99) in DLB, AD, PD	Voxel-level changes in FPCIT binding	Age, MMSE, UPDRS III, duration of symptoms	Spatial normalization and smoothening for voxelwise univariate statistical tests (technique of proportional scaling) with t-statistic for each voxel formed SPM, foci described in terms of spatial extent (k) and peak height (u); Gaussian random field theory; ROC analysis	Decreased bilateral uptake in caudate nucleus, anterior and posterior putamen in DLB and PD versus AD and controls and in AD versus controls; No difference between DLB and PD; ROC AUCs > 0.92
Del Sole et al. [61]	Study the correlations between ^123^I-FP-CIT uptake in the striatum and extrapyramidal signs (EPS) in probable DLB patients	Specific uptake ratio	EPS using UPDRS III score, MMSE	Pearson’s r for correlation	Negative linear correlation between FP-CIT uptake and UPDRS III score in caudate and putamen; Decreased striatal uptake in patients with mild and severe EPS
Donaghy et al. [62]	Compare the neuropsychiatric symptoms and cognitive profile of MCI-LB with MCI-AD	Normal or abnormal FP-CIT SPECT scans	Lewy body Neuropsychiatric Supportive Symptom Count (LBNSSC), calculated based on supportive neuropsychiatric symptoms (non-visual hallucinations, delusions, anxiety, depression, apathy)	T-tests, Mann-Whitney U tests, Chi-squared and Fisher’s exact tests depending on the nature of the data.	Higher symptoms count in MCI-LB than MCI-AD, using a positive FP-CIT SPECT in the list of diagnostic features of MCI-LB
Durcan et al. [63]	Explore whether gastroparesis is early diagnostic marker of prodromal DLB and the relationship with dopaminergic imaging (FP-CIT SPECT)	Visually rated FP-CIT SPECT scans (normal vs abnormal)	Gastroparesis Cardinal Symptom Index (GSCI), MMSE, ACE-R, CDR, CIRS-G	Chi-square, Mann-Whitney, Fisher exact test	No difference in gastroparesis symptom prevalence or severity score and FP-CIT uptake
Gupta et al. [9]	Study the imaging patterns of PCA and DLB with FDG PET/CT and develop a prediction model	Normal vs abnormal TRODAT uptake and ^18^F-FDG PET/CT uptake patterns		Z score analysis, ROC curve analysis, binary logistic regression analysis to determine the OR	Hypometabolism in parieto-temporo-occipital association cortices and cingulate cortices in non-DLB patients; Reduced visual vortex uptake in DLB; Hypometabolism in both groups in occipital association cortex
Hansen et al. [64]	Validating ^123^I-FP-CIT SPECT as a method to diagnose probable DLB in patients with psychiatric symptomatology and suspected DLB	SBR ratios	Clinical and neuropsychological phenotypes via main core and supportive clinical features; supportive biomarkers (EEG slowing posterior, temporal preservation); clinical onset of symptoms (Psych-onset, MCI-onset, Mixed onset)	Fisher’s exact test, Student’s t-tests to compare age groups and number of DLB patients with probable DLB before and after ^123^I-FP-CIT SPECT. Mann-Whitney U test for non-normally distributed group data; ANOVA for core features, clinical features, supportive biomarkers, demographics; two factorial ANOVA for SBR ration between groups (prodromal DLB, possible and probable DLB) as one factor and binary ^123^I-FP CIT SPECT result (nigrostriatal deficit vs no deficit) as the other.	Higher number of probable DLB patients with psychiatric symptoms post-DaTscan than pre-DaTscan; Higher number of prodromal DLB with a psychiatric-phenotype
Huber et al. [10]	Elucidate the link between ^18^F-FDG PET and ^123^I-ioflupane SPECT in the pathophysiological course of dementia with Lewy Bodies	DaT-SPECT Z-Score	Core features of DLB	Pearson’s r for correlation, linear regression analysis (associations between DaT Z-score and FDG-PET SUVr), regression analysis using DaT Z-Scores as predictor and voxel-wise ^18^F-FDG-PET SUVr as outcome variable	Inverse relationship between striatal dopamine availability and relative glucose hypermetabolism in basal ganglia and limbic regions; Increasing dopamine deficiency reflected in metabolic connectivity deteriorations
Iizuka et al. [11]	Investigation of the relationship between awareness of memory-deficit and glucose metabolism in DLB	DaT binding, CIS ratio	Clinical characteristics, educational yearls, MMSE, RAVLT, MAC-Q, Awareness index	Two-sample t-test; Pearson ‘s R for correlations with Bonferroni correction	Decreased Awareness index in DLB than in normal cognition; Association with glucose metabolism in bilateral posterior cingulate cortex and right OFC; No correlation between Awareness index and striatal DAT density
Inagawa et al. [16]	Investigate the efficacy of olfactory (Odor Stick Identification Test for the Japanese) and pareidolia tests (susceptibility to visual hallucinations) to differentiate AD from DLB	SBR on DaTscan, MIBG H/M ratio	OSIT-J and pareidolia test scores, Age, sex, educational history	Mann-Whitney test, Chi-squared, Student’s t-test	Sensitivity/specificity for differentiating DLB from AD: 86/100 for MIBG scintigraphy; 82/96 for SBR uptake; 77/67 for combined OSIT-J and pareidolia test scores; 73/62 for pareidolia test scores; 77/58 for OSIT-J test scores
Iwabuchi et al. [26]	Evaluate the correlation between perfusion SPECT and quantitative indices with DaTscan in patients with DLB, PD, PDD.	Quantitative indices from DaTscan: Specific binding, Putamen-to-caudate, Caudate-to-putamen ratios (SBR, PCR, CPR).	Age, sex, MMSE	Kruskal-Wallis test to compare age, quantitative indices (e.g. SBR) and MMSE between LBD groups (PD, PDD, DLB; post-hoc analysis with Bonferroni correction; Pearson’s chi-square test to compare sex between groups	Correlation between: -decreased PCR and hypoperfusion in the medulla and midbrain; -decreased CPR and hypoperfusion in the right temporoparietal cortex, right precuneus and bilateral temporal cortex; No correlation between decreased SBR index and brain perfusion
Joling et al. [29]	Compare ^123^I-FP-CIT binding to striatal dopamine and the extrastriatal serotonin transporter between PD and DLB	ROI and voxel-based analysis	Age, gender, MMSE	Distribution of data with histograms, Q-Q plots and with Kolmogorov-Smirnov tests; Clinical variables compared with unpaired t-tests or Mann-Whitney U tests as appropriate; ANOVA between PD and DLB for mean binding ration in each ROI	Decreased DAT binding in PD patients than DLB patients (bilateral posterior putamen); Decreased Caudate/putamen ratios in DLB
Joling et al. [30]	Study ^123^I-FP-CIT binding to striatal dopamine and the extrastriatal serotonin transporter between early-stage PD and DLB comparted to healthy controls	ROI and voxel-based analysis	Age, gender, MMSE, UPDRS-III, Hoehn & Yahr	Distribution of data with histograms, Q-Q plots and with Kolmogorov-Smirnov tests; Kruskal-Wallis tests for non-normal distribution. ANCOVA with age as a nuisance covariate.	Decreased DAT binding ratio in both PD and DLB than HC (bilateral caudate head and bilateral posterior putamen); Lower hypothalamic FP-CIT binding ratios in DLB versus HC; Decreased striatal binding in PD and DLB versus HC; Decreased striatal DAT and lower hypothalamic SERT in early-stage PD and early-stage DLB versus HC
Kamagata et al. [31]	Compare SWI with DaT-SPECT for differentiation of DLB from AD/a-MCI	SBR, presence or absence of nigrosome-1 on SWI (visually evaluated)	Age, Sex, disease duration, MMSE	ANOVA with Tukey’s honest significant difference (HSD) test for continuous variables and Chi-square test for categorical variables. Inter-rater variability and reproducibility of visual assessment of SWI and DaTscan with kappa statistic. ANOVA and unpaired t-test to compare contrast ratio and ROI size of nigrosome-1 and assess inter-group differences in SBR for DaTscan. ROC curve analysis for diagnostic utility of normalized signal intensity of nigrosome-1 and SBR from DaTscan, Pearson correlation test for correlations.	Diagnostic accuracy with SWI of 90% (sensitivity: 93%, specificity: 87%) in detecting nigrosome-1 degeneration versus 88.3% (sensitivity: 93%, specificity: 84%) with DaTscan
Kasanuki et al. [32]	Investigate 123I-FP-CIT SPECT findings and clinical relevance in prodromal DLB	Scheltens score on MRI, left and right medial temporal lobe atrophy, occipital hypometabolism, SBR of FP-CIT SPECT	Age, sex, duration of cognitive decline, MMSE, UPDRS-III, medications (Levodopa vs choline esterase inhibitor vs SSRI), Core features (cognitive fluctuations, visual hallucinations and parkinsonism = UPDRS>15), Non-motor symptoms	One-way ANOVA and Student’s t-test for differences in age and duration of disease across the three groups, Chi-square and Fisher’s exact test for differences in categorical data; non-parametric Mann-Whitney and Kruskal-Wallis tests to test for differences. Spearman correlation coefficient for correlations between SBR scores and clinical symptoms in DLB groups.	Decreased mean SBR scores of both prodromal DLB and clinical DLB versus AD; Negative correlations between SBR and UPDRS-III scores in total and clinical DLB groups (not in prodromal DLB); Negative correlation between duration of olfactory dysfunction, RBD and SBR scores in prodromal DLB
Kemp et al.* [50]	Assess the impact of presynaptic dopaminergic imaging with DaTscan SPECT on the clinical diagnosis and subsequent management of patients with possible DLB, referred for imaging	Visually rated DaTscans (normal or abornmal)	Core clinical features (fluctuating cognition, VH, spontaneous parkinsonism, REM sleep disorder, neuroleptic sensitivity, diagnosis of dementia, executive dysfunction, visuospatial dysfunction), Age, sex	NR	Abnormal DaTscan in 20/80 (25%), normal in 60/80 (75%) patients; 18/20 true positives (postscan working clinical diagnosis of DLB) (90%); 58/60 true negatives (alterative clinical diagnosis) (95%); Concordance of DaTscan findings with clinical outcomes in 76/80 cases (95%).
Kobayashi et al. [17]	Evaluate the extent of diagnosis accuracy of combined brain perfusion SPECT, MIBG scintigraphy and DaTscan, and comparison of the 3 tests to determine priority	SBR on DAT-SPECT, early and delayed heart-to-mediastinum ratio on MIBG, regional cerebral blood flow quantified by an automated bp-SPECT analysis program	Age, sex, MMSE, parkinsonism, VH, cognitive fluctuations, REM sleep behavior disorders	Mann-Whitney U-test for relationship between mean MMSE and bp-SPECT, MIBG or DAT-SPECT; Chi-squared or Fishers’ exact test for relationship between clinical features and imaging modalities.	Better sensitivity for MIBG (79%) and Datscan (79%) than bp-SPECT (53%); Higher ratio of patients with RBD in the MIBG-positive; Increased accuracy of diagnosis with the combination of the 3 modalities (Sensitivity: 100%)
Lamotte et al. [33]	Identify if the education level (years of school after first grade) influences cognitive performance and DAT binding in DLB patients	DAT binding in the striatum, caudate nucleus and putamen (primary evaluation criteria)	MMSE score, scores on executive functions, memory and instrumental functions (secondary criteria), motor and non-motor symptoms	Pearson correlation coefficient; Levene’s test for equality of variances for assumption of homogeneity of variance; bi- and multivariate analysis to account for confounding factors	Positive correlation between higher education and DAT binding (putamen and caudate nucleus);
Lim et al. [12]	Optimize the interpretation of ^18^F-FDG-PET images for differentiation of DLB from AD and comparison with DAT imaging	Presence or absence of hypometabolism in the lateral occipital and medial occipital cortices, relative preservation of mid or posterior cingulate region (cingulate island sign).	Age, sex, MMSE, CDR, UPDRS	Sensitivity and specificity for diagnosis of DLB; ANOVA, ROC curve analysis	Higher accuracy and greater size effect for diagnosis of DLB with ^123^I-β-CIT SPECT than ^18^F-FDG-PET
Lloyd et al. [51]	Develop a new visual rating scale for ^123^I-Ioflupane brain imaging in DLB and validate it against autopsy diagnosis	Visual rating scale using the “Newcastle scale” (0 normal, 0.5 very mild/equivocal, 1 mild loss, 2 moderate loss, 3 severe loss)	Clinical diagnosis, autopsy diagnosis	Inter-operator agreement was measured with the intra-class correlation coefficient (ICC, two-way mixed effects models) for each striatal region (right-left caudate and putamen) and for total score, ROC curve analysis for optimal threshold to optimize combined Sn and Sp.	Higher sensitivity/specificity of the Newcastle scale (97%/100%) versus standard scale (97%/80%) with autopsy validation; Inter-rater reliability of Newcastle scale (intra-class correlation coefficient 0.93)
Maltais et al. [52]	Compare three ^123^I-FP-CIT SPECT quantitative methods in patients with neurodegenerative syndromes with neuropathological findings as reference	DQ Striatum, Caudate SBRs (z-score), MIM Striatum, Caudate SBR (z-score), DAT Visual interpretation	Age at scan, time between last scan and death (y), clinical diagnosis before death	ANOVA for continuous variables and Chi-squared test for categorical variables to test for differences amongst the 3 groups (LBD, LBD/AD, no LBD), AUROCs to test for neuropathology discrimination of the semi-quantitative image analysis programs, ICC for assessing the relationship between image analysis program and ROIs, Box-and-whisker plots to display the distribution of ROIs in z-score and SBR format with the relation to neuropathological diagnosis	AUROC values between 0.93 and 1.00 for discrimination between LBD and non-LBD using DaTQUANTm, MIMneuro and manual ROI methods
McKeith et al. [68]	Assess the sensitivity and specificity of ^123^I-FP-CIT SPECT imaging in ante-mortem differentiation of probable DLB from other causes of dementia	Visually interpreted SPECT images (normal or abnormal); abnormal scans subdivided into 3 types: type 1) asymmetric uptake with normal or almost normal putamen activity, type 2) greatly reduced uptake in the putamen on both hemispheres, type 3) virtually absent uptake	Age, sex, UPDRS, Hoehn & Yahr, MMSE, CDR, CAMCOG-R, NPI, Cornelle scale for depression in dementia, clinical assessment of cognitive fluctuations	Chi-squared test for differences among diagnostic groups, ANOVA for normally distributed variables, otherwise non-parametric Kruskal-Wallis test; sensitivity, specificity, positive and negative predictive values for probable and possible DLB according to DATscan diagnosis; Cohen’s kappa statistic for inter-reader agreement	Mean sensitivity of 77.7% of an abnormal DATscan to detect clinically probable DLB; specificity of 90.4% for excluding non-DLB dementia; mean value of 85.7% for overall diagnostic accuracy; PPV of 82.4%, NPV of 87.5%; Inter-reader agreement for rating images was high with kappa coefficient of 0.87
Miyagawa et al. [13]	Asses how well ^123^I-FP-CIT SPECT can differentiate DLB from AD and whether multimodal imaging has additional value	DaTQUANT putamen z-score, PiB-PET global SUVr, FDG-PET CIS ratio	Age, sex, education, core symptoms (RBD, VH, fluctuation score), duration of cognitive decline, UPDRS-III, MMSE	AD and DLB groups compared with Student’s t-test for continuous variables and Chi-squared for categorical variables. Logistic regressions with 1, 2 or 3 modalities as predictors of AD vs DLB. Pearson correlations between continuous imaging biomarkers.	C-statistic of 0.916 with DaTQUANT z-scores of the putamen for differentiating DLB from AD; Added accuracy with multimodal imaging with ^18^F-FDG PET and PiB-PET (c-statistics of 0.968-0.975 adding 1 modality and 0.987-0.996 adding 2 modalities)
Miyamoto et al. [58]	Examine DaTscan in Japanese patients with iRBD as a biomarker for the development of Lewy body disease (PD and DLB)	SBR of striatium (L, R, mean; z-score)	Age, sex, Odor identification test, MMSE, UPDRS-III	Fisher’s exact test and Mann-Whitney U tests for comparison. Wilcoxon matched-pairs signed rank test for changes in SBR and z-score in R, L and average of R and L striatum. ROC curve analysis for cutoff value (Youden’s method). Kaplan-Meier method for plots of estimated proportion of subjects that developed clinically defines LBD over time, compared with log-rank test. Cox proportional hazard for predictive markers, stratified by cutoff values.	Development of LBD in 33.8%; Difference in ROC curve z-score in iRBD patients; Increased risk of LBD if z-score < 2.5 for striatal DAT binding in Kaplan-Meier survival analysis
Morgan et al. [34]	Investigate how well DaTscan differentiates DLB from FTD	Visually rated FP-CIT uptake (0 normal uptake, 1 sligh reduction, 2 significant reduction), dichotomized to normal (scores of 0 or 1) vs abnormal (score of 2)	Sex, age, CDR, CAMCOG-R, MMSE, letter fluency, category fluency, UPDRS, modified Hoehn and Yahr, EPSM (at least one), presence of tremor, rigidity, bradykinesia, VH	Chi-squared, Mann-Whitney and Kruskal-Wallis as appropriate for comparison between groups.	Significant decreased of DAT binding (putamen and caudate) in 9/10 DLB patients; Abnormal DaTscan with reduced DAT (putamen and caudate) in 1/3 of FTD patients; Visually different scans and ROIs between groups
Nakahara et al. [18]	Investigate the relationship between olfaction and frontal lobe cognition using ^123^I-FP-CIT SPECT in PD, PDD or DLB (LBD)	MIBG early and delay, SBR (man, min, average)	Age, Sex, duration of disease, UPDRS, Odor stick identification test score, FAB score	Welch’s t-test for differences between groups (continuous variables). Pairwise comparisons using Chi-squared tests for binary variables. Spearman’s rank correlation coefficients for correlations between pairs of datasets (SBR and FAB scores)	Correlation between OSIT-J scores and SBR in both groups; Correlation between SBR and FAB scores in patients with reduced CBF in frontal lobe (not in normal CBF)
Nicastro et al. [35]	1) Assess the validity of semi-quantitative DaTscan analysis compared to visual analysis in probable DLB and AD; 2) study DLB specific uptake impartment patterns in DLB and correlation of uptake in the presence or absence of parkinsonism	Right and left uptake values via BRASS for caudate, putamen, striatum, as well as Caudate/Putamen (C/P) ratios, striatal asymmetry indices (AIs) for both DLB and AD groups	Age, sex, disease duration, visual assessment (clearly abnormal = stage 1-3	Shapiro-Wilk test to test continuous variables for normality. Non-parametric, two-sample Wilcoxon Rank Sum (Mann-Whitney U) test for VOIs uptake, C/P ratio and striatal AI. Kruskal-Wallis test compare more than two independent groups (DLB with and without parkinsonism and AD), post hoc Mann-Whitney analysis to specifically compare two groups (DLB without parkinsonism and AD).	Abnormal visual staging in 96.8% of DLB patients; Abnormal semi-quantitative analysis in 97.8%; Sensitivity of 100% with combination of visual and semi-quantitative analysis; More pronounced putaminal uptake in DLB patients if associated with parkinsonism
Nicastro et al. [53]	Determine sensitivity of combined visual and semi-quantitative ^123^I-FP-CIT SPECT analysis in a prospective cohort of patients with DLB and degenerative parkinsonisms (PD, MSA, CBS, PSP) to determine the prevalence and clinical significance of Scans Without Evidence of Dopaminergic Deficit (SWEDD).	Semi-quantitative parameters values, visual grading system (0-to-3 system)	Age, sex, disease duration, clinical diagnosis	Shapiro-Wilk test to test continuous variables for normality. Non-parametric, two-sample Wilcoxon Rank Sum (Mann-Whitney U) when appropriate	Normal visual SPECT in only 2.1% of patients with degenerative parkinsonism and 1.9% with DLB; Mild striatal uptake impairment with semi-quantitative analysis in only two patients (1 DLB, 1 PD)
Nicastro et al. [14]	Understanding the metabolic and dopaminergic correlation of presence hallucinations (DH) as well as their relation to a recently defined PH brain network in DLB	Mean caudate nucleus ioflupane uptake, ioflupane SBRs	Age, Sex, education, disease duration, MMSE, UPDRS-III, LEDD, VH	Shapiro-Wilk test to assess continuous variables for normality, t-test of Mann-Whitney U test as appropriate for between-group comparisons, Chi-squared test for discrete variables. Whole brain analysis with a two-sample t-test design (PH+ vs PH-) with ANCOVA taking age and sex as covariates. ROI Analysis with Marsbar toolbox for MATLAB and R: linear mixed-effects model. Seed-to-whole-brain analysis with interregional correlation analysis (IRCA) for both groups.	Decreased ^18^F-FDG uptake in superior frontal and parietal gyri in patients with PH+; Involvement of ventral premotor cortex of PH network with reduced functional connectivity; Negative correlation between ^18^F-FDG vPMC uptake and ^123^I-FP-CIT caudate uptake in PH patients
O’Brien et al. [36]	1) Determine the pattern and clinical correlates of dopamine transporter loss in DLB with Datscan compared with HC and PD, AD or PDD patients; 2) examine whether FP-CIT changes might discriminate between DLB and AD	Main Outcome Measures = Visual ratings of scans and region of interest analysis (binding ratios: mean caudate, anterior and posterior putamen, left and right caudate, anterior and posterior putamen)	Age, Sex, MMSE, CAMCOG, UPDRS-III, duration of illness	Analysis of variance with the Gabriel post hoc tests for normally distributed data, nonparametric Mann-Whitney test. Intersubject variability agreement assessed with Cohen weighted kappa test. Pearson r or Spearman p as appropriate for correlations between clinical and SPECT variables.	Decreased FP-CIT binding in caudate, anterior and posterior putamen in DLB patients versus HC and AD; Good discrimination of DLB and AD with visual and ROI analysis (Sn/Sp 78%/94%, PPV 90%); No difference between DLB, PD and PDD
O’Brien et al.** [37]	Determine the accuracy of ^123^I-FP-CIT SPECT in diagnosing people with possible DLB, i.e. compare the results of visual assessment in probable DLB or non-DLB as determined by the 12-month follow-up diagnosis (consensus panel)	Dichotomized visually rated using 4-point scale (0 normal uptake, 1 unilateral putamen loss, 2 bilateral putamen loss, 3 virtually absent uptake)	MMSE, CAMCOG-R, UPDRS, Hoen and Yahr staging, Neuropsychiatric inventory, VH, Psycholeptics (hypnotic and antipsychotic drugs), Psychoanaleptics (anti-dementia drugs and antidepressants)	Chi-squared tests for differences between probable DLB, possible DLB and non-DLB. ANOVA for normally distributed data. Kruskal-Wallis test if non-normal.	Change of diagnosis from possible to probable DLB in 19/44 (43%) patients and non-DLB in 7/44 (7%); Abnormal baseline scan in 12/19 new probable DLB cases (Sn 63%)
Oliveira et al.*** [59]	Re-evaluate the differentiation of patients with DLB from AD and PD with quantitative analysis of ^123^I-FP-CIT SPECT based on neuropathology diagnoses.	Visual assessment of scans, Semi-quantitative indices	Age, sex, autopsy confirmed diagnosis, clinical diagnosis at baseline	Kruskal-Wallis test for comparison of caudate, putamen binding potentials (CBP, PBP), putamen-to-caudate ratio (PCR) across groups, and post hoc analyses using two-tailed Mann-Whitney U test, correction using the Hold-Bonferroni method.	Decreased CBP and PBP in DLB versus AD patients; Higher PCR in DLB vesus PD patients; Diagnostic accuracies: -Visual rating: 88% in all patients and 96% between PD, AD and DLB -Semi-quantitative: 94% (DLB vs AD), 94% (DLB vs PD vs AD), 93% (DLB, AD, PD vs HC)
Pilotto et al. [38]	Evaluate extra-striatal dopaminergic and serotonergic pathways in PD and DLB with DaTscan	Binding in nigrostriatal and extrastriatal ROIs, SBR in the regions	Age, sex, disease duration, serotonergic/dopaminergic treatments	ANOVA or Mann-Whitney U test for three group (PD vs DLB vs HC) comparisons and two-group (PD vs DLB) comparisons respectively. Chi-squared test for differences in categorical variables. ANOVA with Bonferroni post hoc comparisons adjusted for age and sex for nigrostriatal and extrastriatal FP-CIT SBR values. Post-hoc comparisons between DLB and PD with univariate analysis (adjusted for age, sex, disease duration, SSRI and LEDD).	Decreased ^123^I-FP-CIT SBR in both PD and DLB versus HC in insula, cingulate and thalamus; Decreased ^123^I-FP-CIT SBR in thalamus in DLB versus HC and PD; Correlation between thalamic and cingulate ^123^I-FP-CIT SBR deficits with limbic serotonergic; Correlation between cingulate ^123^I-FP-CIT and widespread cortical monoaminergic projections
Ransmayr et al. [39]	Compare parkinsonian features and loss of striatal dopamine transporter function in DLB and PD	Mean count rates per pixel, striatal (S) to cerebellar (C) ratio, differences between left-right S/C ratios, S/C asymmetry indices	Age, sex, disease duration, UPDRS, CAS, CAI	Kruskal Wallis ANOVA, Mann-Whitney U test, Spearman rank correlation	Decreased S/C ratios in DLB and HC versus PD; Higher total UPDRS scores during practical-off in DLB versus PD; Lower UPDRS extremity subscores in DLB versus PD
Roberts et al. [19]	Provide evidence that MIBG scintigraphy differentiates probable MCI-LB from MCI-AD	Dichotomized cardiac MIBG uptake result (H/M ratio)	Age, sex, BMI, UDRS, MMSE, ACE, ESS, GDS, IADL, CDR, NPI, Memantine, cholinesterase inhibitor, antiparkinsonian drug, fluctuations (baseline), VH (baseline), Parkinsonism (baseline), RBD (baseline)	Levene test, Mann-Whitney U test, Chi-squared test	Diagnosis accuracy with core clinical features: -79% for MIBG (95% CI 68%-87%) -76% for FP-CIT (95% CI 65%-85%)
Roberts et al.**** [54]	Provide evidence of the diagnostic accuracy of dopaminergic imaging at the MCI stage to support or refute its inclusion as a biomarker for MCI with Lewy bodies	SBRs	Age, sex, BMI, UDRS, MMSE, ACE, Epworth Sleepiness scale, Geriatric Depression scale, IADL, CDR, NPI, Memantine, cholinesterase inhibitor, antiparkinsonian drug, fluctuations (baseline), VH (baseline), Parkinsonism (baseline), RBD (baseline)	Student’s t-test or Mann-Whitney U-test; Chi-square; independent samples t-test; Z-scores below -2 calculated; Likelihood ratios from a 2x2 frequency table to estimate the added value of DaTscan	Baseline 123I-FP-CIT visual rating for probable MCI-LB sensitivity of 66%, specificity of 88%, accuracy 76%, positive likelihood ratio 5.3
Roselli et al. [40]	Explore whether 123I-FP-CIT binding in the putamen, caudate nucleus and nucleus accumbens is related to psychiatric symptoms in DLB.	Neuropsychiatric symptoms (delusions, hallucinations, depression, apathy), DAT levels	Age, sex, disease duration, MMSE, CDR, UPDRS-III, NPI, various subscores: hallucinations, delusions, depression, anxiety, apathy, sleep	Spearman’s correlation; Pairwise Pearson’s correlation coefficients; Bonferroni correction.	Inverse correlation between delusions, apathy, depression and DAT levels (caudate);
Sakamoto et al. [20]	Determine whether DAT-SPECT or 123I-MIBG myocardial scintigraphy should be examined first; evaluate superiority of the combined use of DAT-SPECT and MIBG versus either modality alone	SBR, H/M ratio (early and delayed, and washout rate)	Age, sex	ROC analysis with delayed H/M ratio yielding Specificity, sensitivity, accuracy and AUC, 2-sided t test for normally distributed data, 2-sided Mann-Whitney U test for non-normally distributed data	Sensitivity, Specificity and accuracy of diagnosing LBD: - SBR mean with DAT-SPECT: 59.6%, 71.4%, 67.5% - Delayed H/M ratio with MIBG: 85.1%, 91.4% and 88.9% -Combined index: 76.6%, 74.3% and 75.2%
Shimizu et al. [21]	Compare diagnostic value of DAT SPECT vs MIBG myocardial scintigraphy for supporting the diagnosis of DLB and differentiating it from AD; evaluation the use of the combination of the two modalities	SBR, H/M ratio (delayed)	Age, sex, disease duration, length of education, MMSE	Student’s t test, Chi-squared, one-way ANOVA, ROC curve analysis	Sensitivity, Specificity and of differentiating DLB from AD: - DAT-SPECT: 88.2%, 88.9% - Delayed H/M ratio with MIBG: 72.4%, 94.4% -Combined index: 96.1%, 90.7% and higher accuracy than single modality; Higher frequency of parkinsonism in the abnormal DAT SPECT group; Higher frequency of RBD in the abnormal MIBG group.
Shimizu et al. [22]	Compare the diagnostic value of 123I-FP-CIT DAT-SPECT, MRI, perfusion SPECT and MIBG myocardial scintigraphy in differentiating DLB from AD	SBR for DAT-SPECT, H/M ratio (delayed phase) for MIBG, z-scores in the medial occipital lobe for perfusion SPECT, z-scores of hippocampal atrophy for MRI	Age, sex, education, duration of disease, MMSE	Student’s t-test, Chi-squared test, one-way ANOVA, ROC curve analysis	Sensitivity, Specificity and of differentiating DLB from AD: - DAT SPECT: Sn 93.8%, Sp 93.8%), superior accuracy - Delayed H/M ratio with MIBG: Sn 63.5%, Sp 100% - Perfusion SPECT: Sn 71.9%, Sp 59.4% -MRI: Sn 46.9%, Sp 81.3%
Siepel et al. [65]	Explore the clinical course of patients with criteria for clinical DLB but normal FP-CIT SPECT (“false negative”) and patients not fulfilling DLB criteria with an abnormal scan (“false positive”)	Visually rated FP-CIT SPECT	Scores on standardized clinical rating scales for hallucinations, parkinsonism, fluctuations, RBD	Two-step cluster analysis with 4 continuous variables (parkinsonism, hallucinations, cognitive fluctuations and RBD) and log-likelihood.	Increased frequency and severity of parkinsonism and cognitive fluctuation in S+CF- patients (not VH and RBD); Fulfillment of probable DLB criteria at baseline and end of follow-up for S-CF+ patients
Siepel et al. [41]	Explore the association between loss of striatal dopamine transporter binding and DLB symptoms	SBR	UPDRS, NPI, MMSE	Linear regression (DAT SBRs were the dependent variables and cognitive scores the independent variables), corrected for age and sex	Association of dopamine deficiency in DLB with severity of motor symptoms; No correlation between dopamine deficiency and ratings of neurobehavioral disturbances nor overall cognition
Spehl et al. [42]	Evaluate the role of ^123^I-FP-CIT SPECT in the differentiation of DLB, FTD and AD	Binding potential values in caudate nucleus, putamen and whole striatum including caudate/putamen BP ratio and asymmetry indices	Age, sex, symptom duration, MMSE, parkinsonism	Student t-test (continuous data), Chi-squared test (nominal data), ANOVA with post hoc Tukey-Kramer test for cases of multiple group comparisons	Decreased putaminal binding potential in patients with: -DLB versus AD (AUC 0.94) -FTD versus DLB (AUC 0.92); -FTD versus AD (AUC 0.74) Decreased binding potential ration in DLB versus FTD patients (AUC 0.91); High accuracy of combination of putaminal BP and BPR for DLB versus FTD (AUC 0.97); High accuracy in diagnosis of DLB among all patients (AUC 0.95) but but not of FTD (AUC 0.81) and AD (AUC 0.80)
Taylor et al. [43]	Clarify whether chronic ChEi therapy modulates striatal dopamine transporter binding measured by ^123^I-FP-CIT in DLB, AD and PDD patients	Striatal binding (caudate, anterior and posterior putamen)	ChEi use versus non-use, Age, sex, MMSE, severity of parkinsonism and concurrent anti-depressant use, UPDRS-III, duration of illness, time on ChEi for those on medication	Analysis of the effect of ChEi on 123I-FP-CIT SBR with multivariate analysis of covariance (MANCOVA)	Decreased striatal ^123^I-FP-CIT uptake in DLB and PDD versus AD; No significant change for patients with ChEi
Thomas et al. [55]	Investigate the diagnostic value of ^123^I-FP-CIT in a prospective study of a cohort followed up over one year	Visually rated FP-CIT scans (normal or abnormal),	Age, Sex, MMSE, ACE-R, CDR, CIRS-G, IADL, UPDRS, H&Y, ESS, NPI, NPI distress, GDS, Medication at baseline (anti-dementia, -parkinsonian, -psychotic, -depressant)	Chi-squared, t-test and Mann-Whitney for group comparisons; Likelihood ratios for diagnostic value	Visually rated FP-CIT scans to detect: -possible or probable MCI-LB: Sensitivity of 54.2% (95% CI 39.2-68.6), Specificity of 89% (95% CI 70.8-97.6), Likelihood ratio of 4.9; -probable MCI-LB only: Sensitivity: 61% (95% CI 42.5-77.4); -possible MCI-LB only: Sensitivity: 40% (95%CI 16.4-67.7)
Tiraboschi et al. [23]	Compare the diagnostic value of ^123^I-FP-CIT SPECT and MIBG myocardial scintigraphy in differentiating DLB from other dementia subtypes (AD, FTD)	Normal or abnormal visual DaTscans, VOI-based semi-quantitative values	Age, sex, MMSE, CDR, IADL, CIRS severity and comorbidity, CDS, ESS, MFS, Clinical assessment of fluctuations, NPI, North-East Visual Hallucinations Interview	Student’s t-test, Pearson chi-square test for dichotomous variables; comparison of semi-quantitative results between the 2 groups. Sensitivity and specificity determined for both visual and semi-quantitative analyses, as well as PPV, NPV. McNemar test to compare sensitivities and specificities. Cohen kappa statistic for inter-rater agreement for visual assessment.	Sensitivity and specificity for MIBG: 93% and 100%; Sensitivity and specificity for FP-CIT: 90% and 76%; Decreased FP-CIT uptake in 7 non-DLB patients (3 with parkinsonism)
Treglia et al. [24]	Compare myocardial sympathetic imaging using ^123^I-MIBG scintigraphy and striatal dopaminergic imaging using ^123^I-ioflupane (FP-CIT) SPECT in patients with LBD	SBR, H/M ratio	Age, sex	Chi-square for relationship between 2 modalities; Sensitivity, specifitiy, accuracy, PPV and NPV were calculated with 95% confidence interval; McNemar’s test to compare results, Chi-square with Yates’ correction or Fisher’s test when appropriate to assess relationship between MIBG and FP-CIT	MIBG: overall sensitivity of 83%, specificity of 79%, accuracy of 82%, PPV of 86% and NPV of 76%; FP-CIT: 93%, 41%, 73%, 71%, 80%; No difference in the 2 modalities in patients with LBD
Van de Beek et al. [44]	Investigate associations between core and suggestive DLB symptoms and different aspects of disease burden (i.e. IADL, QoL, caregiver burden)	Visual assessments as well as age-matched binding ratio’s of DAT binding	Core and suggestive symptoms, questionnaires for functional activities, QoL, Zarit Caegiver Burden Interview, age, sex, MMSE	Descriptive statistics to characterize core and suggestive features (dichotomized as absent/present), general linear models to evaluate the influence of cognition, core and suggestive symptoms on IADL, univariate and multi-variate models	88% abnormal FP-CIT scans; 95% patients with EEG/MEG abnormalities; 53% patients with a CSF AD profile
Van der Zande et al. [45]	Describe clinical and imaging follow-up of patients with probable DLB with a normal baseline scan (compared to those with abnormal baseline scans)	Binding ratios of FP-CIT SPECT	Usual clinical characteristics	Fisher’s exact test for categorical variables, Mann-Whitney U test for continuous variables, Cohen’s kappa statistic for interobserver variation	7/67 (10.4 %) normally rated FP-CIT scans; Abnormal subsequent control in five DLB/S− patients (average second scan after 1.5 years)
Van der Zande et al. [46]	Study the concomitant AD pathology in DLB on DaTscan and serotonin transporter availability using ^123^I-FP-CIT SPECT	Atrophy corrected ROIs, Binding ratios	CSF biomarker profile	Mann-Whitney U test, Chi-square or Fisher’s exact test as appropriate. Linear regression with Pearson or Spearman correlation between BRs in each ROI (DAT and SERT) and clinical measures (corrected for age and ROI volume)	Decreased FP-CIT binding ratios in the left amygdala (trend in the right hippocampus) in patients with DLB + AD co-pathology; Negative correlation between motor symptoms and striatal DAT binding ratios;
Walker et al. [66]	Determine if detection of dopaminergic degeneration can help distinguish DLB from AD during life	Binding of FP-CIT radioactivity in caudate, anterior and posterior putamen	Age, MMSE, CAMCOG, CDR, BEHAVE-AD, UPDRS, Cornell depression scale, CAPE	ANOVA and t-test were used to assess the difference between the four groups in ipsilateral and contralateral FP-CIT binding in caudate, anterior and posterior putamen and their basic indices; Cohen’s kappa test for inter-rater reliability	Decreased ^123^I-FP-CIT uptake in DLB and PD patients versus AD patients and HC (caudate nucleus, anterior and posterior putamen)
Walker et al. [47]	Compare the patterns of dopaminergic disruption in DLB and PD and evaluate the relationship between extrapyramidal signs and severity of dopaminergic dysfunction	FP-CIT binding (STR/OCC)	MMSE, CAMCOG, CDR, UPDRS, Hoehn and Yahr stage	ANOVA and Student’s t-test for differences between groups of FP-CIT binding in caudate nuclei and anterior and posterior putamen; nonparametric Kruskal-Wallis and Mann-Whitney tests for C/P ratios and asymmetry indices; Spearman’s rank correlation for ordinal data	Decreased ^123^I-FP-CIT striatal binding in DLB and patients versus HC; Decreased binding in DLB versus PD patients in caudate nucleus; Increased asymmetry of uptake in posterior putamen of PD versus DLB patients; Higher mean C/P ratios of PD versus DLB patients and HC
Walker et al. [56]	Determine in a series of dementia patients with autopsy confirmation whether dopaminergic imaging improves accuracy of diagnosis compared to clinical criteria alone.	FP-CIT binding (STR/OCC), visual rating of scans	Family history, rigidity, akinesia, tremor, VH, fluctuations, age, sex, years of education, Hoehn and Yahr stage, MMSe, UPDRS, CAMCOG, CAPE, GDS, CDR, Behave-AD, Neuropathological diagnostic criteria (i.e. neurofibrillary tangles), alpha-synuclein)	Sensitivity and specificity (autopsy = gold standard) of FP-CIT SPECT and of the Consensus DLB criteria (of 1996)	Initial clinical diagnosis of DLB: Sensitivity of 75%, specificity of 42% ^123^I-FP-CIT: Sensitivity of 88%, specificity of 100% Neuropathological diagnosis over 10 years: -8/20 patients DLB -9/20 patients AD (co-existing with cerebrovascular disease) -3/20 patients with other diagnoses
Walker et al. *****[57]	Investigate whether doing a DaTscan in patients with possible DLB would to a more certain diagnosis (probable DLB or non-DLB dementia).	Visual rating (type 1: asymmetric activity, one putamen with reduced uptake; type 2: absent activity of putamen of both hemispheres; type 3: type 2+greatly reduced of absent activity in one or more caudate nuclei)	Primary outcome measure: proportion of patients with a change in clinical diagnosis (to probable DLB or non-DLB) at 8 weeks, secondary outcome was the same at 24 weeks and change in clinician’s confidence of diagnosis at 8 and 24 weeks	Fisher’s exact test; ANCOVA to compare the mean change in clinician’s confidence of diagnosis between baseline and week 8, baseline and week 24 and weeks 8 and 24.	Abnormal scans in 43% of 114 patients; Higher likelihood for clinical change in diagnosis if abnormal scan (82%) versus normal scan (46%)
Ziebell et al. [48]	Identify whether any of the core features of DLB were influenced by disturbances of DAT availability	DAT availability (Non-displaceable binding potential adjusted to age)	Core features of DLB (dementia, hallucinations, fluctuations or parkinsonism)	Unpaired Student’s t-test to compare clinical core symptoms and DAT binding; Linear regression analysis for correlation of continuous data	No correlation between MMSE, Hoehn & Yahr score, fluctuations or hallucinations and striatal DAT availability as measured with ^123^I-PE2I SPECT

*NR* not reported, *MMSE* Folstein Mini-Mental State Examination, *CDR* Clinical Dementia Rating Scale, *CAMCOG-R* Cambridge Cognitive Examination-Revised, *UPDRS* Unified Parkinson’s Disease Rating Scale, *EPSM* extrapyramidal motor signs, *VH* Visual hallucinations, *DQ* DaTQUANT, *FAB* Frontal assessment battery, SBR Striatum-to-Background Ratio, *ROI* region of interest, *ICC* intra-class correlation coefficients, *AUROC* Area under the Receiving operating characteristics, *OSIT-J* Odor stick identification test for the Japanese, *LEDD* Levodopa equivalent daily dosage in mg, *SSRI* selective serotonin reuptake inhibitors, *CAS* clinical asymmetry score, *CAI* clinical asymmetry index, *ACE* Addenbrooke’s Cognitive Examination, *BMI* Body mass index, *CDR* Clinical Dementia Rating, *IADL* instrumental activities of daily life, *NPI* Neuropsychiatric Inventory, *CUSPAD* Columbia University Scale of Psychopathology in Alzheimer’s Disease, *ChEi* Cholinesterase inhibitor, *CIRS* Cumulative Illness Rating Scale, *QoL* Quality of Life, *BEHAVE-AD* bevavioural pathology in Alzheimer’s Disease, *CAPE* Clifton assessment procedure for the elderly

*Kemp et al: 95% change in dx, 94% change in ttt, 93% change in management

**O’Brien et al. [37] : 43% change in diagnosis from possible to probable DLB

***Oliveira: Autopsy diagnosis change in 1/8 normal DaTscans that turned out to be DLB

****Roberts et al. [54]: 42% change in diagnosis from MCI to probable MCI-LB

*****Walker et al. [57]: More patients in the imaging group had a change in diagnosis at 8 and 24 weeks compared with controls (61% versus 4% and 71% versus 16%)

**Table 3:** Methodology and outcome summary of the included studies

Authors	Study objectives	Variables analyzed		Statistical analysis	Outcome summary
		SPECT variables	Clinical variables		
Ceravolo et al. [49]	Assess dopamine transporter function	Ratio of specific (bilateral caudate nucleus, putamen) to non-specific (occipital cortex) radiotracer binding	Age, sex, MMSE, CAMCOG, NPI, UPDRS	NR	Decreased specific to non-specific FP-CIT binding in DLB compared to AD
Chen et al. [15]	Determine clinical phenotypes associated with beta-amyloid PET (A +) and DaT imaging (D +) in MCI-LB (higher risk of progression to probable DLB)	Putamen z score (cutoff ≤ 0.82), beta-amyloid PET PiB SUVR	MMSE, CDR sum of boxes and UPDRS-III scores, visual hallucinations, fluctuations, parkinsonism, probable RBD	T test for continuous variables, Chi-squared test for categorical variables; linear or logistic regression models for predictors (factorial modeling approach using continuous A and D); Age adjusted	Decreased putaminal z score and lower PiB SUVRs independently associated with higher UPDRS-III scores; More probability of RBD in positive DaTscan group
Chiu et al. [60]	Differentiate DLB from AD with motor dysfunction	SBR and visual rating	Novel designed questionnaire, composite scale with motor dysfunction questionnaire (MDQ) and dopamine transporter imaging	Chi-square test for each question in the HAI-MDQ between DLB and non-DLB groups,	Higher frequency of motor dysfunction in DLB group; Lower SBR in DLB group; Sensitivity/Specificity (%): 91/72 (MDQ), 91/80 (SBR), 87/93 (composite scale)
Colloby et al. [25]	Compare diagnostic accuracy of ^99m^Tc-exametazime to ^123^I-FP-CIT for AD and DLB	Normal or Abnormal visual rating of 123I-FP-CIT SPECT Scans (grade 0 = normal, grade 1,2,3 = abnormal)	Age, sex, MMSE, CAMCOG, UPDRS-III, duration of illness	Shapiro–Wilk test for normality of distribution of continuous variables; one-way ANOVA (*F*-Test) for group effects; Nominal data with Chi-square tests; Cohen’s kappa test for inter-observer agreement between raters’ visual assessment; ROC curve analysis for Sn and Sp	Excellent Inter-rater agreement (*κ* = 0.88) for DaTscan; Superior ROC for DaTscan in diagnosing DLB (AUC 0.83, sensitivity: 78.6%, specificity: 87.9) compared to brain perfusion
Colloby et al. [27]	Investigate rate of progression of nigrostriatal dopaminergic loss in DLB, PD, PDD using DaTscan	Striatal binding between groups	Age, MMSE, UPDRS III, duration of symptoms	Shapiro–Wilk test for normality of distribution of continuous variables; Pearson’s r or Spearman’s rho as appropriate for correlations between SPECT and clinical variables; 2-sample dependent t tests for differences in binding rations; ANCOVA (analysis of covariance) for rates of decline in BRs	Faster rates of decline in striatal binding in LBD than controls in caudate nucleus and posterior putamen; Faster rates of decline in striatal binding in DLB & PDD than controls in anterior putamen; Similar rates of decline between DLB, PD, and PDD
Colloby et al. [28]	Investigate the differences in striatal binding using automated statistical parametric mapping (SPM99) in DLB, AD, PD	Voxel-level changes in FPCIT binding	Age, MMSE, UPDRS III, duration of symptoms	Spatial normalization and smoothening for voxel-wise univariate statistical tests (technique of proportional scaling) with t-statistic for each voxel formed SPM, foci described in terms of spatial extent (k) and peak height (u); Gaussian random field theory; ROC analysis	Decreased bilateral uptake in caudate nucleus, anterior and posterior putamen in DLB and PD versus AD and controls and in AD versus controls; No difference between DLB and PD; ROC AUCs > 0.92
Del Sole et al. [61]	Study the correlations between ^123^I-FP-CIT uptake in the striatum and extrapyramidal signs (EPS) in probable DLB patients	Specific uptake ratio	EPS using UPDRS III score, MMSE	Pearson’s r for correlation	Negative linear correlation between FP-CIT uptake and UPDRS III score in caudate and putamen; Decreased striatal uptake in patients with mild and severe EPS
Donaghy et al. [62]	Compare the neuropsychiatric symptoms and cognitive profile of MCI-LB with MCI-AD	Normal or abnormal FP-CIT SPECT scans	Lewy body Neuropsychiatric Supportive Symptom Count (LBNSSC), calculated based on supportive neuropsychiatric symptoms (non-visual hallucinations, delusions, anxiety, depression, apathy)	*T*-tests, Mann–Whitney* U* tests, Chi-squared, and Fisher’s exact tests depending on the nature of the data	Higher symptoms count in MCI-LB than MCI-AD, using a positive FP-CIT SPECT in the list of diagnostic features of MCI-LB
Durcan et al. [63]	Explore whether gastroparesis is early diagnostic marker of prodromal DLB and the relationship with dopaminergic imaging (FP-CIT SPECT)	Visually rated FP-CIT SPECT scans (normal vs abnormal)	Gastroparesis Cardinal Symptom Index (GSCI), MMSE, ACE-R, CDR, CIRS-G	Chi-square, Mann–Whitney, Fisher exact test	No difference in gastroparesis symptom prevalence or severity score and FP-CIT uptake
Gupta et al. [9]	Study the imaging patterns of PCA and DLB with FDG PET/CT and develop a prediction model	Normal vs abnormal TRODAT uptake and ^18^F-FDG PET/CT uptake patterns		Z score analysis, ROC curve analysis, binary logistic regression analysis to determine the OR	Hypometabolism in parieto-temporo-occipital association cortices and cingulate cortices in non-DLB patients; reduced visual vortex uptake in DLB; hypometabolism in both groups in occipital association cortex
Hansen et al. [64]	Validating ^123^I-FP-CIT SPECT as a method to diagnose probable DLB in patients with psychiatric symptomatology and suspected DLB	SBR ratios	Clinical and neuropsychological phenotypes via main core and supportive clinical features; supportive biomarkers (EEG slowing posterior, temporal preservation); clinical onset of symptoms (Psych-onset, MCI-onset, Mixed onset)	Fisher’s exact test, Student’s *t*-tests to compare age groups and number of DLB patients with probable DLB before and after ^123^I-FP-CIT SPECT. Mann–Whitney *U* test for non-normally distributed group data; ANOVA for core features, clinical features, supportive biomarkers, demographics; two factorial ANOVA for SBR ration between groups (prodromal DLB, possible and probable DLB) as one factor and binary ^123^I-FP CIT SPECT result (nigrostriatal deficit vs no deficit) as the other	Higher number of probable DLB patients with psychiatric symptoms post-DaTscan than pre-DaTscan; higher number of prodromal DLB with a psychiatric-phenotype
Huber et al. [10]	Elucidate the link between ^18^F-FDG PET and ^123^I-ioflupane SPECT in the pathophysiological course of dementia with Lewy bodies	DaT-SPECT *Z-*Score	Core features of DLB	Pearson’s r for correlation, linear regression analysis (associations between DaT *Z*-score and FDG-PET SUVr), regression analysis using DaT *Z-*Scores as predictor and voxel-wise ^18^F-FDG-PET SUVr as outcome variable	Inverse relationship between striatal dopamine availability and relative glucose hypermetabolism in basal ganglia and limbic regions; increasing dopamine deficiency reflected in metabolic connectivity deteriorations
Iizuka et al. [11]	Investigation of the relationship between awareness of memory-deficit and glucose metabolism in DLB	DaT binding, CIS ratio	Clinical characteristics, educational yearls, MMSE, RAVLT, MAC-Q, Awareness index	Two-sample *t*-test; Pearson ‘s *R* for correlations with Bonferroni correction	Decreased Awareness index in DLB than in normal cognition; association with glucose metabolism in bilateral posterior cingulate cortex and right OFC; no correlation between Awareness index and striatal DAT density
Inagawa et al. [16]	Investigate the efficacy of olfactory (Odor Stick Identification Test for the Japanese) and pareidolia tests (susceptibility to visual hallucinations) to differentiate AD from DLB	SBR on DaTscan, MIBG H/M ratio	OSIT-J and pareidolia test scores, age, sex, educational history	Mann–Whitney test, Chi-squared, Student’s *t*-test	Sensitivity/specificity for differentiating DLB from AD: 86/100 for MIBG scintigraphy; 82/96 for SBR uptake; 77/67 for combined OSIT-J and pareidolia test scores; 73/62 for pareidolia test scores; 77/58 for OSIT-J test scores
Iwabuchi et al. [26]	Evaluate the correlation between perfusion SPECT and quantitative indices with DaTscan in patients with DLB, PD, PDD	Quantitative indices from DaTscan: specific binding, Putamen-to-caudate, Caudate-to-putamen ratios (SBR, PCR, CPR)	Age, sex, MMSE	Kruskal–Wallis test to compare age, quantitative indices (e.g., SBR) and MMSE between LBD groups (PD, PDD, DLB; post-hoc analysis with Bonferroni correction; Pearson’s chi-square test to compare sex between groups	Correlation between: -decreased PCR and hypoperfusion in the medulla and midbrain; -decreased CPR and hypoperfusion in the right temporoparietal cortex, right precuneus and bilateral temporal cortex; no correlation between decreased SBR index and brain perfusion
Joling et al. [29]	Compare ^123^I-FP-CIT binding to striatal dopamine and the extrastriatal serotonin transporter between PD and DLB	ROI and voxel-based analysis	Age, gender, MMSE	Distribution of data with histograms, Q-Q plots and with Kolmogorov–Smirnov tests; clinical variables compared with unpaired *t*-tests or Mann–Whitney *U* tests as appropriate; ANOVA between PD and DLB for mean binding ration in each ROI	Decreased DAT binding in PD patients than DLB patients (bilateral posterior putamen); decreased Caudate/putamen ratios in DLB
Joling et al. [30]	Study ^123^I-FP-CIT binding to striatal dopamine and the extrastriatal serotonin transporter between early-stage PD and DLB compared to healthy controls	ROI and voxel-based analysis	Age, gender, MMSE, UPDRS-III, Hoehn & Yahr	Distribution of data with histograms, Q-Q plots and with Kolmogorov–Smirnov tests; Kruskal–Wallis tests for non-normal distribution. ANCOVA with age as a nuisance covariate	Decreased DAT binding ratio in both PD and DLB than HC (bilateral caudate head and bilateral posterior putamen); Lower hypothalamic FP-CIT binding ratios in DLB versus HC; decreased striatal binding in PD and DLB versus HC; Decreased striatal DAT and lower hypothalamic SERT in early-stage PD and early-stage DLB versus HC
Kamagata et al. [31]	Compare SWI with DaT-SPECT for differentiation of DLB from AD/a-MCI	SBR, presence or absence of nigrosome-1 on SWI (visually evaluated)	Age, sex, disease duration, MMSE	ANOVA with Tukey’s honest significant difference (HSD) test for continuous variables and Chi-square test for categorical variables. Inter-rater variability and reproducibility of visual assessment of SWI and DaTscan with kappa statistic. ANOVA and unpaired *t*-test to compare contrast ratio and ROI size of nigrosome-1 and assess inter-group differences in SBR for DaTscan. ROC curve analysis for diagnostic utility of normalized signal intensity of nigrosome-1 and SBR from DaTscan, Pearson correlation test for correlations	Diagnostic accuracy with SWI of 90% (sensitivity: 93%, specificity: 87%) in detecting nigrosome-1 degeneration versus 88.3% (sensitivity: 93%, specificity: 84%) with DaTscan
Kasanuki et al. [32]	Investigate 123I-FP-CIT SPECT findings and clinical relevance in prodromal DLB	Scheltens score on MRI, left and right medial temporal lobe atrophy, occipital hypometabolism, SBR of FP-CIT SPECT	Age, sex, duration of cognitive decline, MMSE, UPDRS-III, medications (Levodopa vs choline esterase inhibitor vs SSRI), Core features (cognitive fluctuations, visual hallucinations and parkinsonism = UPDRS > 15), Non-motor symptoms	One-way ANOVA and Student’s *t*-test for differences in age and duration of disease across the three groups, Chi-square and Fisher’s exact test for differences in categorical data; non-parametric Mann–Whitney and Kruskal–Wallis tests to test for differences. Spearman correlation coefficient for correlations between SBR scores and clinical symptoms in DLB groups	Decreased mean SBR scores of both prodromal DLB and clinical DLB versus AD; Negative correlations between SBR and UPDRS-III scores in total and clinical DLB groups (not in prodromal DLB); Negative correlation between duration of olfactory dysfunction, RBD and SBR scores in prodromal DLB
Kemp et al.* [50]	Assess the impact of presynaptic dopaminergic imaging with DaTscan SPECT on the clinical diagnosis and subsequent management of patients with possible DLB, referred for imaging	Visually rated DaTscans (normal or abornmal)	Core clinical features (fluctuating cognition, VH, spontaneous parkinsonism, REM sleep disorder, neuroleptic sensitivity, diagnosis of dementia, executive dysfunction, visuospatial dysfunction), Age, sex	NR	Abnormal DaTscan in 20/80 (25%), normal in 60/80 (75%) patients; 18/20 true positives (postscan working clinical diagnosis of DLB) (90%); 58/60 true negatives (alterative clinical diagnosis) (95%); Concordance of DaTscan findings with clinical outcomes in 76/80 cases (95%)
Kobayashi et al. [17]	Evaluate the extent of diagnosis accuracy of combined brain perfusion SPECT, MIBG scintigraphy and DaTscan, and comparison of the 3 tests to determine priority	SBR on DAT-SPECT, early and delayed heart-to-mediastinum ratio on MIBG, regional cerebral blood flow quantified by an automated bp-SPECT analysis program	Age, sex, MMSE, parkinsonism, VH, cognitive fluctuations, REM sleep behavior disorders	Mann–Whitney *U*-test for relationship between mean MMSE and bp-SPECT, MIBG or DAT-SPECT; Chi-squared or Fishers’ exact test for relationship between clinical features and imaging modalities	Better sensitivity for MIBG (79%) and Datscan (79%) than bp-SPECT (53%); Higher ratio of patients with RBD in the MIBG-positive; increased accuracy of diagnosis with the combination of the 3 modalities (sensitivity: 100%)
Lamotte et al. [33]	Identify if the education level (years of school after first grade) influences cognitive performance and DAT binding in DLB patients	DAT binding in the striatum, caudate nucleus and putamen (primary evaluation criteria)	MMSE score, scores on executive functions, memory and instrumental functions (secondary criteria), motor and non-motor symptoms	Pearson correlation coefficient; Levene’s test for equality of variances for assumption of homogeneity of variance; bi- and multivariate analysis to account for confounding factors	Positive correlation between higher education and DAT binding (putamen and caudate nucleus);
Lim et al. [12]	Optimize the interpretation of ^18^F-FDG-PET images for differentiation of DLB from AD and comparison with DAT imaging	Presence or absence of hypometabolism in the lateral occipital and medial occipital cortices, relative preservation of mid or posterior cingulate region (cingulate island sign)	Age, sex, MMSE, CDR, UPDRS	Sensitivity and specificity for diagnosis of DLB; ANOVA, ROC curve analysis	Higher accuracy and greater size effect for diagnosis of DLB with ^123^I-β-CIT SPECT than ^18^F-FDG-PET
Lloyd et al. [51]	Develop a new visual rating scale for ^123^I-Ioflupane brain imaging in DLB and validate it against autopsy diagnosis	Visual rating scale using the “Newcastle scale” (0 normal, 0.5 very mild/equivocal, 1 mild loss, 2 moderate loss, 3 severe loss)	Clinical diagnosis, autopsy diagnosis	Inter-operator agreement was measured with the intra-class correlation coefficient (ICC, two-way mixed effects models) for each striatal region (right-left caudate and putamen) and for total score, ROC curve analysis for optimal threshold to optimize combined Sn and Sp	Higher sensitivity/specificity of the Newcastle scale (97%/100%) versus standard scale (97%/80%) with autopsy validation; Inter-rater reliability of Newcastle scale (intra-class correlation coefficient 0.93)
Maltais et al. [52]	Compare three ^123^I-FP-CIT SPECT quantitative methods in patients with neurodegenerative syndromes with neuropathological findings as reference	DQ Striatum, Caudate SBRs (*z*-score), MIM Striatum, Caudate SBR (*z*-score), DAT visual interpretation	Age at scan, time between last scan and death (y), clinical diagnosis before death	ANOVA for continuous variables and Chi-squared test for categorical variables to test for differences amongst the 3 groups (LBD, LBD/AD, no LBD), AUROCs to test for neuropathology discrimination of the semi-quantitative image analysis programs, ICC for assessing the relationship between image analysis program and ROIs, Box-and-whisker plots to display the distribution of ROIs in *z*-score and SBR format with the relation to neuropathological diagnosis	AUROC values between 0.93 and 1.00 for discrimination between LBD and non-LBD using DaTQUANTm, MIMneuro, and manual ROI methods
Miyagawa et al. [13]	Asses how well ^123^I-FP-CIT SPECT can differentiate DLB from AD and whether multimodal imaging has additional value	DaTQUANT putamen* z*-score, PiB-PET global SUVr, FDG-PET CIS ratio	Age, sex, education, core symptoms (RBD, VH, fluctuation score), duration of cognitive decline, UPDRS-III, MMSE	AD and DLB groups compared with Student’s *t*-test for continuous variables and Chi-squared for categorical variables. Logistic regressions with 1, 2, or 3 modalities as predictors of AD vs DLB. Pearson correlations between continuous imaging biomarkers	C-statistic of 0.916 with DaTQUANT *z*-scores of the putamen for differentiating DLB from AD; added accuracy with multimodal imaging with ^18^F-FDG PET and PiB-PET (c-statistics of 0.968–0.975 adding 1 modality and 0.987–0.996 adding 2 modalities)
Miyamoto et al. [58]	Examine DaTscan in Japanese patients with iRBD as a biomarker for the development of Lewy body disease (PD and DLB)	SBR of striatium (L, R, mean; *z*-score)	Age, sex, Odor identification test, MMSE, UPDRS-III	Fisher’s exact test and Mann–Whitney *U* tests for comparison. Wilcoxon matched-pairs signed rank test for changes in SBR and* z*-score in R, L and average of R and L striatum. ROC curve analysis for cutoff value (Youden’s method). Kaplan–Meier method for plots of estimated proportion of subjects that developed clinically defines LBD over time, compared with log-rank test. Cox proportional hazard for predictive markers, stratified by cutoff values	Development of LBD in 33.8%; Difference in ROC curve *z*-score in iRBD patients; Increased risk of LBD if *z-*score < 2.5 for striatal DAT binding in Kaplan–Meier survival analysis
Morgan et al. [34]	Investigate how well DaTscan differentiates DLB from FTD	Visually rated FP-CIT uptake (0 normal uptake, 1 slight reduction, 2 significant reduction), dichotomized to normal (scores of 0 or 1) vs abnormal (score of 2)	Sex, age, CDR, CAMCOG-R, MMSE, letter fluency, category fluency, UPDRS, modified Hoehn and Yahr, EPSM (at least one), the presence of tremor, rigidity, bradykinesia, VH	Chi-squared, Mann–Whitney, and Kruskal–Wallis as appropriate for comparison between groups	Significant decreased of DAT binding (putamen and caudate) in 9/10 DLB patients; abnormal DaTscan with reduced DAT (putamen and caudate) in 1/3 of FTD patients; visually different scans and ROIs between groups
Nakahara et al. [18]	Investigate the relationship between olfaction and frontal lobe cognition using ^123^I-FP-CIT SPECT in PD, PDD, or DLB (LBD)	MIBG early and delay, SBR (man, min, average)	Age, sex, duration of disease, UPDRS, odor stick identification test score, FAB score	Welch’s *t*-test for differences between groups (continuous variables). Pairwise comparisons using Chi-squared tests for binary variables. Spearman’s rank correlation coefficients for correlations between pairs of datasets (SBR and FAB scores)	Correlation between OSIT-J scores and SBR in both groups; correlation between SBR and FAB scores in patients with reduced CBF in frontal lobe (not in normal CBF)
Nicastro et al. [35]	1) Assess the validity of semi-quantitative DaTscan analysis compared to visual analysis in probable DLB and AD; 2) study DLB specific uptake impartment patterns in DLB and correlation of uptake in the presence or absence of parkinsonism	Right and left uptake values via BRASS for caudate, putamen, striatum, as well as Caudate/Putamen (C/P) ratios, striatal asymmetry indices (AIs) for both DLB and AD groups	Age, sex, disease duration, visual assessment (clearly abnormal = stage 1–3	Shapiro–Wilk test to test continuous variables for normality. Non-parametric, two-sample Wilcoxon Rank Sum (Mann–Whitney *U*) test for VOIs uptake, C/P ratio and striatal AI. Kruskal–Wallis test compare more than two independent groups (DLB with and without parkinsonism and AD), post hoc Mann–Whitney analysis to specifically compare two groups (DLB without parkinsonism and AD)	Abnormal visual staging in 96.8% of DLB patients; abnormal semi-quantitative analysis in 97.8%; Sensitivity of 100% with combination of visual and semi-quantitative analysis; more pronounced putaminal uptake in DLB patients if associated with parkinsonism
Nicastro et al. [53]	Determine sensitivity of combined visual and semi-quantitative ^123^I-FP-CIT SPECT analysis in a prospective cohort of patients with DLB and degenerative parkinsonisms (PD, MSA, CBS, PSP) to determine the prevalence and clinical significance of Scans Without Evidence of Dopaminergic Deficit (SWEDD)	Semi-quantitative parameters values, visual grading system (0-to-3 system)	Age, sex, disease duration, clinical diagnosis	Shapiro–Wilk test to test continuous variables for normality. Non-parametric, two-sample Wilcoxon Rank Sum (Mann–Whitney *U*) when appropriate	Normal visual SPECT in only 2.1% of patients with degenerative parkinsonism and 1.9% with DLB; mild striatal uptake impairment with semi-quantitative analysis in only two patients (1 DLB, 1 PD)
Nicastro et al. [14]	Understanding the metabolic and dopaminergic correlation of presence hallucinations (DH) as well as their relation to a recently defined PH brain network in DLB	Mean caudate nucleus ioflupane uptake, ioflupane SBRs	Age, Sex, education, disease duration, MMSE, UPDRS-III, LEDD, VH	Shapiro–Wilk test to assess continuous variables for normality, *t*-test of Mann–Whitney *U* test as appropriate for between-group comparisons, Chi-squared test for discrete variables. Whole brain analysis with a two-sample *t*-test design (PH + vs PH-) with ANCOVA taking age and sex as covariates. ROI Analysis with Marsbar toolbox for MATLAB and R: linear mixed-effects model. Seed-to-whole-brain analysis with interregional correlation analysis (IRCA) for both groups	Decreased ^18^F-FDG uptake in superior frontal and parietal gyri in patients with PH + ; involvement of ventral premotor cortex of PH network with reduced functional connectivity; negative correlation between ^18^F-FDG vPMC uptake and ^123^I-FP-CIT caudate uptake in PH patients
O’Brien et al. [36]	1) Determine the pattern and clinical correlates of dopamine transporter loss in DLB with Datscan compared with HC and PD, AD, or PDD patients; 2) examine whether FP-CIT changes might discriminate between DLB and AD	Main outcome measures = Visual ratings of scans and region of interest analysis (binding ratios: mean caudate, anterior and posterior putamen, left and right caudate, anterior and posterior putamen)	Age, Sex, MMSE, CAMCOG, UPDRS-III, duration of illness	Analysis of variance with the Gabriel post hoc tests for normally distributed data, nonparametric Mann–Whitney test. Intersubject variability agreement assessed with Cohen weighted kappa test. Pearson *r* or Spearman p as appropriate for correlations between clinical and SPECT variables	Decreased FP-CIT binding in caudate, anterior and posterior putamen in DLB patients versus HC and AD; good discrimination of DLB and AD with visual and ROI analysis (Sn/Sp 78%/94%, PPV 90%); No difference between DLB, PD and PDD
O’Brien et al.** [37]	Determine the accuracy of ^123^I-FP-CIT SPECT in diagnosing people with possible DLB, i.e., compare the results of visual assessment in probable DLB or non-DLB as determined by the 12-month follow-up diagnosis (consensus panel)	Dichotomized visually rated using 4-point scale (0 normal uptake, 1 unilateral putamen loss, 2 bilateral putamen loss, 3 virtually absent uptake)	MMSE, CAMCOG-R, UPDRS, Hoen and Yahr staging, Neuropsychiatric inventory, VH, psycholeptics (hypnotic and antipsychotic drugs), psychoanaleptics (anti-dementia drugs and antidepressants)	Chi-squared tests for differences between probable DLB, possible DLB and non-DLB. ANOVA for normally distributed data. Kruskal–Wallis test if non-normal	Change of diagnosis from possible to probable DLB in 19/44 (43%) patients and non-DLB in 7/44 (7%); abnormal baseline scan in 12/19 new probable DLB cases (Sn 63%)
Oliveira et al.*** [59]	Re-evaluate the differentiation of patients with DLB from AD and PD with quantitative analysis of ^123^I-FP-CIT SPECT based on neuropathology diagnoses	Visual assessment of scans, Semi-quantitative indices	Age, sex, autopsy confirmed diagnosis, clinical diagnosis at baseline	Kruskal–Wallis test for comparison of caudate, putamen binding potentials (CBP, PBP), putamen-to-caudate ratio (PCR) across groups, and post hoc analyses using two-tailed Mann–Whitney *U* test, correction using the Hold-Bonferroni method	Decreased CBP and PBP in DLB versus AD patients; higher PCR in DLB versus PD patients; diagnostic accuracies: -visual rating: 88% in all patients and 96% between PD, AD and DLB -Semi-quantitative: 94% (DLB vs AD), 94% (DLB vs PD vs AD), 93% (DLB, AD, PD vs HC)
Pilotto et al. [38]	Evaluate extra-striatal dopaminergic and serotonergic pathways in PD and DLB with DaTscan	Binding in nigrostriatal and extrastriatal ROIs, SBR in the regions	Age, sex, disease duration, serotonergic/dopaminergic treatments	ANOVA or Mann–Whitney *U* test for three group (PD vs DLB vs HC) comparisons and two-group (PD vs DLB) comparisons respectively. Chi-squared test for differences in categorical variables. ANOVA with Bonferroni post hoc comparisons adjusted for age and sex for nigrostriatal and extrastriatal FP-CIT SBR values. Post-hoc comparisons between DLB and PD with univariate analysis (adjusted for age, sex, disease duration, SSRI, and LEDD)	Decreased ^123^I-FP-CIT SBR in both PD and DLB versus HC in insula, cingulate and thalamus; Decreased ^123^I-FP-CIT SBR in thalamus in DLB versus HC and PD; Correlation between thalamic and cingulate ^123^I-FP-CIT SBR deficits with limbic serotonergic; correlation between cingulate ^123^I-FP-CIT and widespread cortical monoaminergic projections
Ransmayr et al. [39]	Compare parkinsonian features and loss of striatal dopamine transporter function in DLB and PD	Mean count rates per pixel, striatal (S) to cerebellar (C) ratio, differences between left–right S/C ratios, S/C asymmetry indices	Age, sex, disease duration, UPDRS, CAS, CAI	Kruskal Wallis ANOVA, Mann–Whitney *U* test, Spearman rank correlation	Decreased S/C ratios in DLB and HC versus PD; higher total UPDRS scores during practical-off in DLB versus PD; lower UPDRS extremity subscores in DLB versus PD
Roberts et al. [19]	Provide evidence that MIBG scintigraphy differentiates probable MCI-LB from MCI-AD	Dichotomized cardiac MIBG uptake result (H/M ratio)	Age, sex, BMI, UDRS, MMSE, ACE, ESS, GDS, IADL, CDR, NPI, Memantine, cholinesterase inhibitor, antiparkinsonian drug, fluctuations (baseline), VH (baseline), Parkinsonism (baseline), RBD (baseline)	Levene test, Mann–Whitney *U* test, Chi-squared test	Diagnosis accuracy with core clinical features: -79% for MIBG (95% CI 68–87%) -76% for FP-CIT (95% CI 65–85%)
Roberts et al.**** [54]	Provide evidence of the diagnostic accuracy of dopaminergic imaging at the MCI stage to support or refute its inclusion as a biomarker for MCI with Lewy bodies	SBRs	Age, sex, BMI, UDRS, MMSE, ACE, Epworth Sleepiness scale, Geriatric Depression scale, IADL, CDR, NPI, Memantine, cholinesterase inhibitor, antiparkinsonian drug, fluctuations (baseline), VH (baseline), Parkinsonism (baseline), RBD (baseline)	Student’s *t*-test or Mann–Whitney *U*-test; Chi-square; independent samples *t*-test; *Z*-scores below − 2 calculated; Likelihood ratios from a 2 × 2 frequency table to estimate the added value of DaTscan	Baseline 123I-FP-CIT visual rating for probable MCI-LB sensitivity of 66%, specificity of 88%, accuracy 76%, positive likelihood ratio 5.3
Roselli et al. [40]	Explore whether 123I-FP-CIT binding in the putamen, caudate nucleus and nucleus accumbens is related to psychiatric symptoms in DLB	Neuropsychiatric symptoms (delusions, hallucinations, depression, apathy), DAT levels	Age, sex, disease duration, MMSE, CDR, UPDRS-III, NPI, various subscores: hallucinations, delusions, depression, anxiety, apathy, sleep	Spearman’s correlation; Pairwise Pearson’s correlation coefficients; Bonferroni correction	Inverse correlation between delusions, apathy, depression, and DAT levels (caudate);
Sakamoto et al. [20]	Determine whether DAT-SPECT or 123I-MIBG myocardial scintigraphy should be examined first; evaluate superiority of the combined use of DAT-SPECT and MIBG versus either modality alone	SBR, H/M ratio (early and delayed, and washout rate)	Age, sex	ROC analysis with delayed H/M ratio yielding Specificity, sensitivity, accuracy and AUC, 2-sided t test for normally distributed data, 2-sided Mann–Whitney *U* test for non-normally distributed data	Sensitivity, Specificity and accuracy of diagnosing LBD: - SBR mean with DAT-SPECT: 59.6%, 71.4%, 67.5% - Delayed H/M ratio with MIBG: 85.1%, 91.4%, and 88.9% -Combined index: 76.6%, 74.3%, and 75.2%
Shimizu et al. [21]	Compare diagnostic value of DAT SPECT vs MIBG myocardial scintigraphy for supporting the diagnosis of DLB and differentiating it from AD; evaluation the use of the combination of the two modalities	SBR, H/M ratio (delayed)	Age, sex, disease duration, length of education, MMSE	Student’s *t* test, Chi-squared, one-way ANOVA, ROC curve analysis	Sensitivity, Specificity and of differentiating DLB from AD: - DAT-SPECT: 88.2%, 88.9% - Delayed H/M ratio with MIBG: 72.4%, 94.4% -Combined index: 96.1%, 90.7% and higher accuracy than single modality; higher frequency of parkinsonism in the abnormal DAT SPECT group; higher frequency of RBD in the abnormal MIBG group
Shimizu et al. [22]	Compare the diagnostic value of 123I-FP-CIT DAT-SPECT, MRI, perfusion SPECT and MIBG myocardial scintigraphy in differentiating DLB from AD	SBR for DAT-SPECT, H/M ratio (delayed phase) for MIBG, *z*-scores in the medial occipital lobe for perfusion SPECT, *z*-scores of hippocampal atrophy for MRI	Age, sex, education, duration of disease, MMSE	Student’s *t*-test, Chi-squared test, one-way ANOVA, ROC curve analysis	Sensitivity, Specificity and of differentiating DLB from AD: - DAT SPECT: Sn 93.8%, Sp 93.8%), superior accuracy - Delayed H/M ratio with MIBG: Sn 63.5%, Sp 100% - Perfusion SPECT: Sn 71.9%, Sp 59.4% -MRI: Sn 46.9%, Sp 81.3%
Siepel et al. [65]	Explore the clinical course of patients with criteria for clinical DLB but normal FP-CIT SPECT (“false negative”) and patients not fulfilling DLB criteria with an abnormal scan (“false positive”)	Visually rated FP-CIT SPECT	Scores on standardized clinical rating scales for hallucinations, parkinsonism, fluctuations, RBD	Two-step cluster analysis with 4 continuous variables (parkinsonism, hallucinations, cognitive fluctuations and RBD) and log-likelihood	Increased frequency and severity of parkinsonism and cognitive fluctuation in S + CF- patients (not VH and RBD); fulfillment of probable DLB criteria at baseline and end of follow-up for S-CF + patients
Siepel et al. [41]	Explore the association between loss of striatal dopamine transporter binding and DLB symptoms	SBR	UPDRS, NPI, MMSE	Linear regression (DAT SBRs were the dependent variables and cognitive scores the independent variables), corrected for age and sex	Association of dopamine deficiency in DLB with severity of motor symptoms; no correlation between dopamine deficiency and ratings of neurobehavioral disturbances nor overall cognition
Spehl et al. [42]	Evaluate the role of ^123^I-FP-CIT SPECT in the differentiation of DLB, FTD, and AD	Binding potential values in caudate nucleus, putamen and whole striatum including caudate/putamen BP ratio and asymmetry indices	Age, sex, symptom duration, MMSE, parkinsonism	Student *t*-test (continuous data), Chi-squared test (nominal data), ANOVA with post hoc Tukey–Kramer test for cases of multiple group comparisons	Decreased putaminal binding potential in patients with: -DLB versus AD (AUC 0.94) -FTD versus DLB (AUC 0.92); -FTD versus AD (AUC 0.74) Decreased binding potential ration in DLB versus FTD patients (AUC 0.91); High accuracy of combination of putaminal BP and BPR for DLB versus FTD (AUC 0.97); High accuracy in diagnosis of DLB among all patients (AUC 0.95) but not of FTD (AUC 0.81) and AD (AUC 0.80)
Taylor et al. [43]	Clarify whether chronic ChEi therapy modulates striatal dopamine transporter binding measured by ^123^I-FP-CIT in DLB, AD and PDD patients	Striatal binding (caudate, anterior and posterior putamen)	ChEi use versus non-use, Age, sex, MMSE, severity of parkinsonism and concurrent anti-depressant use, UPDRS-III, duration of illness, time on ChEi for those on medication	Analysis of the effect of ChEi on 123I-FP-CIT SBR with multivariate analysis of covariance (MANCOVA)	Decreased striatal ^123^I-FP-CIT uptake in DLB and PDD versus AD; no significant change for patients with ChEi
Thomas et al. [55]	Investigate the diagnostic value of ^123^I-FP-CIT in a prospective study of a cohort followed up over one year	Visually rated FP-CIT scans (normal or abnormal),	Age, sex, MMSE, ACE-R, CDR, CIRS-G, IADL, UPDRS, H&Y, ESS, NPI, NPI distress, GDS, Medication at baseline (anti-dementia, -parkinsonian, -psychotic, -depressant)	Chi-squared, *t*-test and Mann–Whitney for group comparisons; likelihood ratios for diagnostic value	Visually rated FP-CIT scans to detect: -possible or probable MCI-LB: sensitivity of 54.2% (95% CI 39.2–68.6), specificity of 89% (95% CI 70.8–97.6), likelihood ratio of 4.9; -probable MCI-LB only: Sensitivity: 61% (95% CI 42.5–77.4); -possible MCI-LB only: Sensitivity: 40% (95%CI 16–4–67.7)
Tiraboschi et al. [23]	Compare the diagnostic value of ^123^I-FP-CIT SPECT and MIBG myocardial scintigraphy in differentiating DLB from other dementia subtypes (AD, FTD)	Normal or abnormal visual DaTscans, VOI-based semi-quantitative values	Age, sex, MMSE, CDR, IADL, CIRS severity and comorbidity, CDS, ESS, MFS, clinical assessment of fluctuations, NPI, North-East Visual Hallucinations Interview	Student’s *t*-test, Pearson chi-square test for dichotomous variables; comparison of semi-quantitative results between the 2 groups. Sensitivity and specificity determined for both visual and semi-quantitative analyses, as well as PPV, NPV. McNemar test to compare sensitivities and specificities. Cohen kappa statistic for inter-rater agreement for visual assessment	Sensitivity and specificity for MIBG: 93% and 100%; sensitivity and specificity for FP-CIT: 90% and 76%; decreased FP-CIT uptake in 7 non-DLB patients (3 with parkinsonism)
Treglia et al. [24]	Compare myocardial sympathetic imaging using ^123^I-MIBG scintigraphy and striatal dopaminergic imaging using ^123^I-ioflupane (FP-CIT) SPECT in patients with LBD	SBR, H/M ratio	Age, sex	Chi-square for relationship between 2 modalities; sensitivity, specifitiy, accuracy, PPV, and NPV were calculated with 95% confidence interval; McNemar’s test to compare results, Chi-square with Yates’ correction or Fisher’s test when appropriate to assess relationship between MIBG and FP-CIT	MIBG: overall sensitivity of 83%, specificity of 79%, accuracy of 82%, PPV of 86% and NPV of 76%; FP-CIT: 93%, 41%, 73%, 71%, 80%; No difference in the 2 modalities in patients with LBD
Van de Beek et al. [44]	Investigate associations between core and suggestive DLB symptoms and different aspects of disease burden (i.e., IADL, QoL, caregiver burden)	Visual assessments as well as age-matched binding ratio’s of DAT binding	Core and suggestive symptoms, questionnaires for functional activities, QoL, Zarit Caegiver Burden Interview, age, sex, MMSE	Descriptive statistics to characterize core and suggestive features (dichotomized as absent/present), general linear models to evaluate the influence of cognition, core and suggestive symptoms on IADL, univariate and multi-variate models	88% abnormal FP-CIT scans; 95% patients with EEG/MEG abnormalities; 53% patients with a CSF AD profile
Van der Zande et al. [45]	Describe clinical and imaging follow-up of patients with probable DLB with a normal baseline scan (compared to those with abnormal baseline scans)	Binding ratios of FP-CIT SPECT	Usual clinical characteristics	Fisher’s exact test for categorical variables, Mann–Whitney *U* test for continuous variables, Cohen’s kappa statistic for interobserver variation	7/67 (10.4%) normally rated FP-CIT scans; abnormal subsequent control in five DLB/S − patients (average second scan after 1.5 years)
Van der Zande et al. [46]	Study the concomitant AD pathology in DLB on DaTscan and serotonin transporter availability using ^123^I-FP-CIT SPECT	Atrophy corrected ROIs, binding ratios	CSF biomarker profile	Mann–Whitney *U* test, Chi-square or Fisher’s exact test as appropriate. Linear regression with Pearson or Spearman correlation between BRs in each ROI (DAT and SERT) and clinical measures (corrected for age and ROI volume)	Decreased FP-CIT binding ratios in the left amygdala (trend in the right hippocampus) in patients with DLB + AD co-pathology; negative correlation between motor symptoms and striatal DAT binding ratios;
Walker et al. [66]	Determine if detection of dopaminergic degeneration can help distinguish DLB from AD during life	Binding of FP-CIT radioactivity in caudate, anterior and posterior putamen	Age, MMSE, CAMCOG, CDR, BEHAVE-AD, UPDRS, Cornell depression scale, CAPE	ANOVA and *t*-test were used to assess the difference between the four groups in ipsilateral and contralateral FP-CIT binding in caudate, anterior and posterior putamen and their basic indices; Cohen’s kappa test for inter-rater reliability	Decreased ^123^I-FP-CIT uptake in DLB and PD patients versus AD patients and HC (caudate nucleus, anterior and posterior putamen)
Walker et al. [47]	Compare the patterns of dopaminergic disruption in DLB and PD and evaluate the relationship between extrapyramidal signs and severity of dopaminergic dysfunction	FP-CIT binding (STR/OCC)	MMSE, CAMCOG, CDR, UPDRS, Hoehn and Yahr stage	ANOVA and Student’s *t*-test for differences between groups of FP-CIT binding in caudate nuclei and anterior and posterior putamen; nonparametric Kruskal–Wallis and Mann–Whitney tests for C/P ratios and asymmetry indices; Spearman’s rank correlation for ordinal data	Decreased ^123^I-FP-CIT striatal binding in DLB and patients versus HC; decreased binding in DLB versus PD patients in caudate nucleus; Increased asymmetry of uptake in posterior putamen of PD versus DLB patients; higher mean C/P ratios of PD versus DLB patients and HC
Walker et al. [56]	Determine in a series of dementia patients with autopsy confirmation whether dopaminergic imaging improves accuracy of diagnosis compared to clinical criteria alone	FP-CIT binding (STR/OCC), visual rating of scans	Family history, rigidity, akinesia, tremor, VH, fluctuations, age, sex, years of education, Hoehn and Yahr stage, MMSe, UPDRS, CAMCOG, CAPE, GDS, CDR, Behave-AD, Neuropathological diagnostic criteria (i.e., neurofibrillary tangles), alpha-synuclein)	Sensitivity and specificity ( autopsy = gold standard) of FP-CIT SPECT and of the Consensus DLB criteria (of 1996)	Initial clinical diagnosis of DLB: Sensitivity of 75%, specificity of 42% ^123^I-FP-CIT: sensitivity of 88%, specificity of 100% Neuropathological diagnosis over 10 years: -8/20 patients DLB -9/20 patients AD (co-existing with cerebrovascular disease) -3/20 patients with other diagnoses
Walker et al. *****[57]	Investigate whether doing a DaTscan in patients with possible DLB would to a more certain diagnosis (probable DLB or non-DLB dementia)	Visual rating (type 1: asymmetric activity, one putamen with reduced uptake; type 2: absent activity of putamen of both hemispheres; type 3: type 2 + greatly reduced of absent activity in one or more caudate nuclei)	Primary outcome measure: proportion of patients with a change in clinical diagnosis (to probable DLB or non-DLB) at 8 weeks, secondary outcome was the same at 24 weeks and change in clinician’s confidence of diagnosis at 8 and 24 weeks	Fisher’s exact test; ANCOVA to compare the mean change in clinician’s confidence of diagnosis between baseline and week 8, baseline and week 24 and weeks 8 and 24	Abnormal scans in 43% of 114 patients; Higher likelihood for clinical change in diagnosis if abnormal scan (82%) versus normal scan (46%)
Ziebell et al. [48]	Identify whether any of the core features of DLB were influenced by disturbances of DAT availability	DAT availability (Non-displaceable binding potential adjusted to age)	Core features of DLB (dementia, hallucinations, fluctuations or parkinsonism)	Unpaired Student’s *t*-test to compare clinical core symptoms and DAT binding; Linear regression analysis for correlation of continuous data	No correlation between MMSE, Hoehn & Yahr score, fluctuations or hallucinations and striatal DAT availability as measured with ^123^I-PE2I SPECT

^*^Kemp et al.: 95% change in dx, 94% change in ttt, 93% change in management

^**^O’Brien et al. (2009): 43% change in diagnosis from possible to probable DLB

^***^Oliveira: Autopsy diagnosis change in 1/8 normal DaTscans that turned out to be DLB

^****^Roberts et al. (2021): 42% change in diagnosis from MCI to probable MCI-LB

^*****^Walker et al. (2015): More patients in the imaging group had a change in diagnosis at 8 and 24 weeks compared with controls (61% versus 4% and 71% versus 16%)

*NR* not reported, *MMSE* Folstein Mini-Mental State Examination, *CDR* Clinical Dementia Rating Scale, *CAMCOG-R* Cambridge Cognitive Examination-Revised, *UPDRS* Unified Parkinson’s Disease Rating Scale, *EPSM* extrapyramidal motor signs, *VH* visual hallucinations, *DQ* DaTQUANT, *FAB* Frontal assessment battery, *SBR* striatum-to-background ratio, *ROI* region of interest, *ICC* intra-class correlation coefficients, *AUROC* area under the receiving operating characteristics, *OSIT-J* odor stick identification test for the Japanese, *LEDD* Levodopa equivalent daily dosage in mg, *SSRI* selective serotonin reuptake inhibitors, *CAS* clinical asymmetry score, *CAI* clinical asymmetry index, *ACE* Addenbrooke’s Cognitive Examination, *BMI* body mass index, *CDR* clinical dementia rating, *IADL* instrumental activities of daily life, *NPI* neuropsychiatric inventory, *CUSPAD* Columbia University Scale of Psychopathology in Alzheimer’s Disease, *ChEi* cholinesterase inhibitor, *CIRS* Cumulative Illness Rating Scale, *QoL* quality of life, *BEHAVE-AD* behavioral pathology in Alzheimer’s disease, *CAPE* Clifton assessment procedure for the elderly

### Qualitative analysis (systematic review)

#### Basic study and patient characteristics

Using the database search, 59 full-text articles reporting on the diagnostic performance of functional dopaminergic scintigraphic imaging in the diagnosis of Lewy body dementia were selected (Supplementary Table 1) [9–68].

All 59 selected articles were published within the last 21 years (32 were published since 2017 with the new DLB criteria). Several countries from Europe, North America and Asia were represented. 46% (27/59) of the studies were retrospective and 42% (25/59) were prospective. (< 1% Case–control/cohort *n* = 4, cross-sectional *n* = 4). Most (81%) of the articles were single-center studies (48/59).

In 17 out of 59 studies, functional dopaminergic scintigraphic imaging was investigated as the single imaging modality in patients with DLB, whereas in the remaining studies, functional dopaminergic scintigraphic imaging was performed in addition to ^18^F-FDG PET (*n* = 6) [9–14], ß-Amyloid PET (*n* = 1) [15], metaiodobenzylguanidine myocardial scintigraphy (MIBG) (*n* = 9) [16–24], brain perfusion SPECT imaging with ^99m^technetium-exametazime (n = 1) [25], ^99m^technetium-ethyl cysteinate dimer (*n* = 1) [17], and N-isopropylp-[^123^I] iodoamphetamine (*n* = 1) [26], 44% (*n* = 26) of the studies included patients with LBD only, while a mixed patient population with different types of dementia were included in the rest of the studies, in particular Alzheimer’s disease (AD) (*n* = 33), frontotemporal dementia (FTD) (*n* = 8), corticobasal syndrome (CBS) (*n* = 4), multi-system atrophy (MSA) (*n* = 2), progressive supranuclear palsy (PSP) (*n* = 2), Creutzfeldt-Jakob disease (CJD) (*n* = 1), vascular dementia (VD) (*n* = 3), vascular parkinsonism (VP) (*n* = 2), and normal pressure hydrocephalus (NPH) (*n* = 1).

Only a few studies (*n* = 31) reported disease duration [9, 10, 12–14, 18, 21, 22, 25, 27–48].

The mean patient age was 74 years and ranged from 64 to 82 years. The mean percentage of male patients was approximately 60%.

The diagnostic performance of dopamine transporter imaging in the assessment of nigrostriatal function loss was investigated in 19 studies (32%) in patients with DLB [17, 19, 20, 22, 23, 27, 35–37, 45, 49–57], while the differentiation of DLB from other entities in the Lewy body disease spectrum (DLB, PD, PDD) was assessed in 15 studies (25%) [18, 24, 26, 27, 29, 30, 35, 37–39, 43, 47, 53, 58, 59]. The correlation of dopamine transporter imaging to clinical phenotypes, core symptoms and clinical scores in DLB was addressed in 19 studies (32%) [11, 14–16, 18, 32, 33, 35, 40, 41, 44, 48, 58, 60–65]. Other studies compared the outcome of functional dopaminergic scintigraphic imaging to perfusion SPECT (rCBF) or ^18^F-FDG PET/CT (*n* = 8) [9, 10, 12, 13, 17, 25, 26, 49] or MIBG myocardial scintigraphy (*n* = 7) [17, 20–24, 54]. A total of 31% (*n* = 18) of the studies investigated the role of functional dopaminergic scintigraphic imaging in differentiating DLB from other types of dementia [12, 13, 21–23, 31, 34, 35, 37, 42, 42, 43, 49, 54, 59, 62, 66, 67]. The comparison of striatal dopamine receptor binding with extrastriatal serotonin transporter binding was the subject of 4 studies only (7%) [29, 30, 38, 46].

#### Technical aspects

Heterogeneous technical aspects were found among the included studies (Table 2). The radiotracer used was [^123^I]N‑ω‑fluoropropyl‑2β‑carbomethoxy‑3β‑(4‑iodophenyl) nortropane (^123^I-FP-CIT) in 54 studies (91.5%) [all others], 2 beta-carboxymethoxy-3 beta-(4-iodophenyl)tropane (^123^I-ß-CIT) in 2 studies (3.4%) [12, 39], ^123^I-N-(3-iodoprop-2E-enyl)-2-b-carbomethoxy-3b-(4-methylphenyl) nortropane (^123^I-PE2I) in one study (1.7%) [48], and Technetium-99 m labeled tropane derivative (^99m^Tc-TRODAT-1) in 2 studies (3.4%) [9, 60]. The hybrid imaging modality was SPECT/CT was used in 20 studies [9–11, 14, 15, 17, 19, 20, 22, 26, 29–31, 33, 35, 37, 52, 54, 58, 60], while SPECT alone was used in 29 studies [16, 18, 23–25, 34, 37–45, 47–51, 55, 56, 62–66, 68] and the combination of SPECT and MRI was used in 11 studies [13, 27–30, 32, 46, 53, 57, 60, 61]. The reported mean injected activity of radiolabelled ^123^I-FP-CIT ranged from 110 to 210 MBq (in absolute values). The time interval between radiotracer injection and image acquisition varied among studies, ranging from 2 to 6 h after injection. The duration of acquisition of images also varied among studies for FP-CIT, ranging from 24 to 60 min. Some studies (*n* = 27) did not report one or more of the aforementioned technical aspects.

Image analysis was performed using visual analysis in 9 studies (15%), semi-quantitative analysis 29 studies (49%) and a combination of semi-quantitative and visual analysis in 16 studies (27%) (Table 2). Briefly, semi-quantitative analysis is the quantification of specific binding ratios using the occipital lobe for intensity normalization from off-target binding of the radiotracer.

### Main findings

#### DLB versus other dementia and LBD spectrum

The clinical differentiation of DLB from other forms of dementia like AD can be challenging, as clinical features may overlap and co-pathologies often occur [69, 70]. Multiple studies confirm that dopaminergic imaging can help distinguish DLB from AD [13, 21, 22, 35–37, 49, 59, 66].

##### DLB versus AD

Dopamine transport (DAT) imaging can help distinguish DLB from AD in vivo through the measure of specific binding ratios (SBRs) of the radioligand. The specific (i.e., bilateral caudate nuclei, putamen) to non-specific (i.e., occipital cortex) FP-CIT binding ratio in DLB patients is lower than in AD patients [49]. Uptake of FP-CIT in the putamen is significantly lower in patients with DLB compared to those with AD, and discordant cases (i.e., AD patients with very low putamen uptake) exist but often show mixed LB and AD pathologies in post-mortem neuropathological confirmation studies [13]. Compared to patients with AD, patients with DLB have reduced FP-CIT binding on all levels of the striatum, i.e., caudate nucleus, anterior and posterior putamen [36]. With regards to laterality, when analyzed using the mean right and left SBRs, FP-CIT uptake is markedly lower in patients with DLB as compared to patients with AD [21].

In addition to distinguishing DLB from AD, DAT SPECT imaging also allows the distinction of DLB from amnestic mild cognitive impairment, considered by some as a prodromal stage of AD with an accuracy of 88% [31]. Further, some authors provide evidence for distinguishing mild cognitive impairment associated DLB from that associated with AD [54, 62]

##### DLB versus FTD

Frontotemporal degeneration (FTD) can be difficult to distinguish from DLB by visual rating of FP-CIT alone [34]. However, semi-quantitative assessment of the putaminal binding and the binding ratio of FP-CIT, as well as the combination of these two parameters provides high accuracy to distinguish DLB from FTD (AUC 0.92, 0.91 and 0.97 respectively) [42]. Tiraboschi et al. recognize the possibility to rule out dementia subtypes like FTD and progressive supranuclear palsy (PSP) using DAT imaging as well as MIBG myocardial scintigraphy. They recognized in all such patients that striatal FP-CIT uptake was reduced, whereas uptake of ^123^I-MIBG was normal [23].

##### DLB versus PSP

In accordance with previous knowledge in the literature, PSP has a markedly decreased striatal DAT and a uniform involvement in the caudate and putamen [71, 72], but this is when comparing PSP to PD and MSA, not DLB, and is thus outside the scope of our systematic review.

##### DLB versus PD and PDD

There are considerable clinical and pathological similarities between dementia with Lewy bodies (DLB) and idiopathic Parkinson’s disease (PD). However, dopaminergic SPECT imaging may identify differences in patterns of dopaminergic deficit between each entity. For instance, Walker et al. showed that DLB patients have lower FP-CIT binding in the caudate nucleus than PD patients, and that PD patients have a greater asymmetry of uptake in the posterior putamen, confirming a selective pattern of dopaminergic degeneration in both entities (i.e., degeneration of ventrolateral nigral neurons in PD) [47].

Parkinson’s disease with dementia (PDD) shares very similar clinical and cognitive features with DLB. Colloby et al. performed serial FP-CIT SPECT studies, which found similar rates of dopaminergic loss in DLB, PD and PDD [27].

With regards to DLB versus PD, Ransmayr et al. found that DLB presented with more severe loss of dopaminergic transporter function than PD [39].

FP-CIT SPECT has a low specificity in differentiating PD and DLB from other degenerative parkinsonian syndromes, i.e., atypical parkinsonian syndromes like multisystem atrophy (MSA), corticobasal degeneration (CBD) and progressive supranuclear palsy (PSP), as they all demonstrate striatal dopaminergic deficits [24]. In a prospective analysis, Nicastro et al. confirmed this as visual and semi-quantitative assessment of FP-CIT SPECT is normal in only a negligible proportion of patients with DLB and other degenerative parkinsonian syndromes [35].

#### Correlation of DAT imaging with clinical presentations & scores

##### Dopaminergic imaging and parkinsonism

The association between parkinsonian symptoms (i.e., extra-pyramidal motor symptoms like rigidity, brady-/akinesia) and FP-CIT uptake has been studied but results are controversial: some authors [48] found no significant difference between striatal dopamine transport availability and severity of motor parkinsonism measured by the Hoehn and Yahr score in DLB patients, whereas others like Siepel et al. did [41].

Chiu et al. demonstrated that a motor dysfunction questionnaire (MDQ) used to distinguish characteristic parkinsonian features of DLB patients positively correlates with the Unified Parkinson Disease Rating Scale motor scale (UPDRS-m) as well as with dopamine transporter imaging [60]. A composite scale of MDQ and visual rating of DaTscans is more accurate to distinguish DLB from AD or healthy controls than DaTscan or the MDQ questionnaire alone (see Table 3). The UPDRS-m also inversely correlates with FP-CIT uptake in the caudate and the putamen, and patients with even mild extra-pyramidal symptoms had similarly less abnormal FP-CIT uptake than those with severe parkinsonism [61]. Nicastro et al. showed that patients with DLB with parkinsonism features had more pronounced dysfunction of putaminal uptake versus a diffuse pattern and higher uptake values in patients with DLB and without parkinsonism features [35].

In summary, despite the initial ambivalence in the literature, these data show that dopaminergic scintigraphic imaging can correlate with the presence and severity of motor parkinsonism in DLB patients, even at early stages of symptoms, when used in combination of clinical scores and questionnaires. More importantly, parkinsonism in DLB can be highlighted through specific patterns of radiotracer uptake.

##### Prodromal DLB

Mild cognitive impairment (MCI) with one or more core features of DLB (fluctuations of attention and cognitive impairment, visual hallucinations, rapid eye-movement (REM) sleep behavior disorder (RBD), and parkinsonism) corresponds to the prodromal phase of DLB that may be present several years before a clinical diagnosis, referred as MCI with Lewy bodies (MCI-LB) [68]. A prospective longitudinal case study carried out over 2 to 5 years by Siepel et al. showed that visually assessed FP-CIT SPECT detects patients with DLB before they develop the complete clinical syndrome, and that the frequency and severity of parkinsonism and cognitive fluctuations increased during the follow-up period [65]. Other research criteria for prodromal DLB include psychiatric-onset DLB and delirium-onset DLB, but these entities have not been extensively studied with regards to dopaminergic imaging and are not included in the scope of this review [73].

##### Neuropsychiatric symptoms (cognition, awareness and hallucinations)

Neuropsychiatric presentation of patients is a key feature in dementia with Lewy bodies. Visual hallucinations are the psychiatric symptoms that are included among the core clinical features of DLB, but other neuropsychiatric features are now considered as supportive features, such as non-visual hallucinations such as presence hallucinations, delusions, depression, and anxiety [3]. Donaghy et al. compared prodromal DLB and AD patients and showed that MCI-LB patients were four times more likely than MCI-AD patients to present two or more of the five supportive neuropsychiatric symptoms. [62]. However, studies that show a link between dopaminergic imaging and neuropsychiatric symptoms are scarce [74]. Nonetheless, evidence shows that the onset of symptoms in DLB patients who show dopaminergic dysfunction through positive FP-CIT SPECT occurs more often with psychiatric symptoms than cognitive impairment. Furthermore, it is generally accepted that the neuropsychological profile of DLB patients will show impacted attentional, executive and visuospatial deficits with relatively preserved episodic memory, unlike AD [64]. When episodic memory is affected in DLB patients, it suggests the presence of a concomitant AD [74]. Although less prevalent in patients with DLB than AD, Iizuka et al. showed that the awareness of memory-deficits in DLB patients, measured by the discrepancy between subjective and objective memory scores, is more impaired than in patients with normal cognition. Interestingly, the awareness index does not correlate with striatal DAT density, but does with hypometabolism of cortical midline structures (i.e., bilateral occipital and parietal association cortices, bilateral temporal cortex, precuneus, and posterior cingulate cortex) shown by ^18^F-FDG-PET [11].

Reduced FP-CIT SPECT binding is useful in predicting the development of LBD within five years in patients presenting with isolated or idiopathic RBD (iRBD), as shown by Kaplan–Meier survival analysis by Miyamoto and colleagues [58]. Important non-visual hallucinations that DLB patients frequently present are presence hallucinations (PH), corresponding to a vivid sensation of somebody nearby in the absence of any physical person [14]. PH occurs frequently in PD, especially at early stages [75–77]. Nicastro et al. showed that DLB patients with PH have widespread frontoparietal ^18^F-FDG hypometabolism, and that ^18^F-FDG uptake in the ventral premotor cortex (vPMC) is negatively correlated with FP-CIT uptake in the caudate nucleus. As for visual hallucinations (VH), Roselli et al. have reported that FP-CIT uptake is inversely associated with their severity and frequency [40]. Among other non-motor symptoms associated with DLB, duration of olfactory dysfunction negatively correlates with striatal specific binding ratios of FP-CIT SPECT [32]. Furthermore, clinical scores that test olfactory decline and susceptibility to visual hallucinations, the odor stick identification and pareidolia tests respectively, can aid in differentiating DLB from AD, albeit less sensitive and specific than FP-CIT uptake [16] (see Table 3). In a more recent study, Nakahara et al. showed that olfactory dysfunction correlates with lower FP-CIT binding independently of cerebral blood flow in the frontal lobe (assessed through perfection SPECT), unlike clinically assessed frontal lobe dysfunction which only showed a negative correlation in patients with frontal lobe hypoperfusion [18].

In DLB patients, a higher level of education is associated with better scores in neuropsychological tests that assess visuoconstructive functions and retrieval strategies, and correlates with higher dopamine transporter binding in the striatum, caudate nucleus and putamen bilaterally [33].

#### Diagnostic performance of DAT imaging and other modalities

A retrospective analysis assessing the impact of dopamine transporter imaging on patients with suspected DLB during their diagnostic workup showed significant impact on diagnosis and subsequent management, as 90% of patient with an abnormal DaTscan had a postscan clinical diagnosis of DLB, and 95% of patients with normal imaging had an alternative clinical diagnosis [50]. Similarly, a randomized multi-center trial by Walker et al. showed that DAT imaging significantly helps clinicians change their diagnosis from possible DLB to probable DLB [57].

Patients who meet clinical criteria for DLB but have a normal DaTscan remain a challenge. In this context, a retrospective study from the Amsterdam Dementia Cohort [45] found that in almost all DLB patients with negative DaTscans, a follow-up ^123^I-FP-CIT SPECT (average 1.5 years after first DaTscan) was abnormal emphasizing the importance of repeating DaTscans if the clinical diagnosis is difficult.

There seems to be a benefit in combining visual and semi-quantitative assessments to discriminate between DLB and AD patients, with a combined sensitivity of 100% [35]. Oliveira et al. computed the bihemispheric caudate binding potentials (CBP), putamen binding potentials (PBP) and putamen-to-caudate ratios (PCR) (derived from the ratio of mean counts across voxels of the regions of interest over the mean counts across voxels of the background reference region), finding that DLB patients had lower CBP and PBPs than AD patients and higher PCR than PD patients, providing an accuracy of 94% in classifying DLB versus AD and DLB versus PD [59].

The use of other technical methods to measure FP-CIT binding, such as through the use of software packages for brain imaging analyses (e.g., Statistical Parametrical Mapping), has been shown to have comparable discriminatory power as visual rating [28].

Multiple studies compared the diagnostic accuracy of dopaminergic imaging using FP-CIT against perfusion SPECT/PET modalities, or their combined use. In comparison to dopaminergic transporter imaging, ^18^F-FDG PET imaging was less accurate and had a lower effect size, but regional hypometabolism in the lateral occipital cortex can be used to exclude the diagnosis of DLB, and the so-called “cingulate island sign” (relative preservation of the mid or posterior cingulate gyrus) is very specific to DLB [12]. Huber et al. showed an inverse relationship between FP-CIT uptake and glucose metabolism in the basal ganglia and limbic regions, referred to relative glucose hypermetabolism [10]. Other tracers such as ^99m^Tc-exametazime also has lower accuracy than FP-CIT in distinguishing between AD and DLB (AUC of 0.64 and 0.83) [25]. However, this radiotracer can identify selective occipital hypoperfusion on LBD, as compared to decreased temporo-parietal blood flow of AD [49]. Other studies confirm these regional differences in hypometabolism, i.e., reduced ^18^F-FDG PET uptake in the visual cortex in DLB patients, and specific decreased blood flow in parieto-temporo-occipital association cortices in a form of AD [9].

Patients in the LBD spectrum have regional reduction in striatal FP-CIT uptake and changes in brain perfusion, as measured by ^123^I-IMP SPECT, such that decreased putamen-to-caudate ration correlates with hypoperfusion in the brainstem whereas decreased caudate-to-putamen ratio correlates with right temporal cortex hypoperfusion [26].

Cardiac ^123^I-metaiodobenzylguanidine sympathetic innervation imaging (MIBG) is included in the diagnostic criteria in the most recent consensus criteria for DLB [3] as an indicative biomarker. Overall, FP-CIT SPECT and MIBG myocardial scintigraphy have similar diagnostic accuracies when distinguishing DLB from other dementias, although FP-CIT SPECT has the highest sensitivity [24]. Other studies show that MIBG scintigraphy is more specific for excluding non-DLB dementias and is particularly useful when the only core feature exhibited by the patient is parkinsonism [23]. Concerning the prodromal stage of DLB, MIBG myocardial scintigraphy has a specificity, sensitivity and accuracy of 59%, 88%, and 75%, respectively, to distinguish patients with MCI-LB from those with MCI-AD [54].

Multimodal imaging shows high accuracy in diagnosing DLB. Miyagawa et al. demonstrated almost perfect areas under the curve (AUC), ranging from 0.987 to 0.996, in differentiating DLB from AD when using FP-SPECT combined with ^18^F-FDG PET and ^11^C-Pittsburgh compound B (PiB)-PET [13]. It also seems useful to combine dopamine transporter imaging, myocardial scintigraphy and brain-perfusion SPECT for the diagnosis of DLB, which yields a sensitivity of 100% [17]. Sakamoto et al. show that MIBG myocardial scintigraphy alone is superior (sensitivity, specificity and accuracy of 85, 91%, and 89%) to a combined index of FP-CIT SPECT and MIBG SPECT (76.6%, 74.3%, and 75.2%) [20]. Another study by Shimizu et al. compared the diagnostic performance of FP-CIT SPECT, MIBG, perfusion SPECT, and MRI (for quantification of atrophy), and found that FP-CIT SPECT is the most accurate modality overall (sensitivity and specificity of 93.8% and 93.8%, respectively) to differentiate between DLB and AD, and MIBG myocardial scintigraphy has low sensitivity but high specificity (62.5% and 100%, respectively) [22]. Combining DAT SPECT and MIBG myocardial scintigraphy surpass the accuracy of either modalities alone according to some authors [21].

Finally, amyloid PET imaging, using the ^11^C-Pittsburgh compound B (PiB) radiotracer, has been compared to standard FP-CIT imaging and do not have a higher diagnostic accuracy (measured by AUC) to distinguish DLB from AD [13]. In MCI-LB, it is possible to study the co-existence of β-amyloid pathology through Amyloid PET, but the phenotype of both β-amyloid positive and FP-CIT positive is rare, as the majority of the studied MCI-LB patients have decreased dopaminergic activity and low β-amyloid deposition [15]. Comparative performance between more recent AmyPET radiotracers such as flutemetamol and florbetapir and FP-CIT imaging has not been studied in the literature.

#### DAT versus SERT

FP-CIT has affinity to both dopamine (DAT) and serotonin (SERT) transporters, therefore it is possible to image both the integrity of dopaminergic striatal and serotoninergic extrastriatal systems simultaneously [30]. However, extrastriatal serotonin transporter (SERT) is seldom studied along with striatal dopaminergic transporter (DAT) binding using FP-CIT SPECT imaging. Some authors found no difference in extrastriatal SERT binding between DLB and PD patients using FP-CIT [29], but others showed that only DLB patients had impairments in serotoninergic pathways of the thalamus [38]. Further, Joling et al. showed that DLB patients have lower hypothalamic SERT availability as compared to standard reference [30]. Finally, Van der Zande et al. studied DLB patients with concomitant AD pathology (defined with cerebrospinal fluid tau/aβ-42 ratio) and found that these patients had lower extrastriatal FP-CIT SERT binding in limbic brain regions (i.e., left amygdala) [46].

Medication such as chronic cholinesterase inhibitors (ChEi) do not influence the radioligand’s binding to striatal DATs therefore do not influence the diagnostic performance of ^123^I-FP-CIT imaging [43]. The authors took into account the effect of selective serotonin reuptake inhibitors (SSRIs) like citalopram and paroxetine on striatal FP-CIT binding, increasing its availability: they had similar proportions of subjects taking antidepressants in those taking ChEi and those without ChEi. The interaction between serotonin and dopamine systems in the striatum is of interest since depression is one of the prodromal symptoms of DLB and AD, and the use of SSRIs is thus frequent in these patients.

## Discussion

### Summary of evidence

The present study is an updated systematic literature review involving 59 primary studies, constituting the largest collection of studies relating to the diagnosis of dementia with Lewy bodies using scintigraphic dopaminergic imaging to this day. Based on the body of evidence that was hereby studied, the use of dopamine transporter imaging provides support in the diagnosis of DLB from other forms of dementia, and within the larger spectrum of Lewy body diseases. Dopaminergic scintigraphic imaging enables accurate discrimination between DLB and AD. As for other forms of neurodegenerative parkinsonian syndromes such as FTD, PSP, and CBD, semi-quantitative measures of DAT uptake cannot clearly differentiate them from DLB. Within the spectrum of Lewy body disease, some patterns of FP-CIT uptake (i.e., lower FP-CIT binding in the caudate nucleus in DLB than PD patients and greater asymmetry of uptake in the posterior putamen with degeneration of ventrolateral nigral in PD patients) have been proposed to specifically identify PD from DLB and PDD, whereas discriminating between the latter two is more challenging. Whether this is due to the different modalities of pharmacological treatments and the patients’ clinical response remains unclear and requires further investigation. Similarly, we could ask ourselves what effects cognitive fluctuations have on FP-CIT binding. Clear patterns of radioligand uptake can be identified using semi-quantitative and/or simple visual rating, and this can be done in prodromal stages of dementia. There is solid evidence to consider motor symptoms and parkinsonism, measured by the validated clinical scores, as adjunct factors to FP-CIT SPECT imaging. The same goes for non-motor symptoms, especially behavioral symptoms. These clinical variables greatly aid the diagnostic accuracy of functional imaging, even at the prodromal stage of DLB. Specifically for the neuropsychiatric symptoms that DLB initially present, such as visual and non-visual hallucinations, relevance of FP-CIT SPECT imaging in the early stages of the disease exists and has been shown in only a few studies, and further investigations are required. For instance, it is unclear whether patterns of ligand uptake can be differentially identified for patients presenting major or minor hallucinations, as the methods of classifying and reporting of these symptoms is not standardized and has been insufficiently studied with regards to dopaminergic scintigraphic imaging.

DAT imaging can be complemented by other imaging modalities, namely by myocardial MIBG scintigraphy, brain-perfusion SPECT and ^18^F-FDG-PET. Essentially, MIBG myocardial scintigraphy is more specific than DAT SPECT imaging, whereas the latter is more sensitive in detecting DLB. FDG-PET can be used to highlight certain signs that are highly specific to DLB, such as the relative preservation of the posterior cingulate (cingulate island sign*)* and occipital hypometabolism. Combinations of striatal scintigraphy, as well as brain-perfusion SPECT and FDG-PET can identify regional correlations of hypoperfusion and striatal DAT availability and ascertain the diagnosis of DLB with greater sensitivity and specificity.

**Fig. 3:**
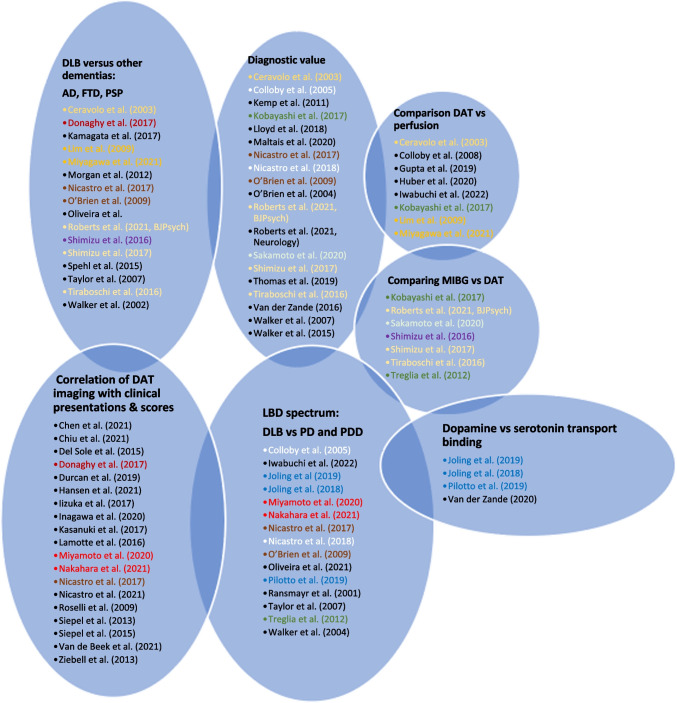
Venn diagram of selected studies according to their main outcomes (color-coded for overlapping studies)

The current review updates the meta-analysis performed by Nihashi et al. in 2018, which itself was an update of their 2015 meta-analysis [6, 78]. Since we did not perform a meta-analysis, we did not compare specificities and sensitivities with these previous studies. Admittedly, we considered there to be too much heterogeneity in the studied populations and subsequent imperfection in comparing reference results. However, our review adds 23 new studies, all but two (*n* = 21) including the use of semi-quantitative assessment. In the study by Nihashi and colleagues, semi-quantitative image analysis was still relatively new, thus limiting the number of analyzed articles. In our review, we propose groups of study findings that are pooled according to their main outcomes (see Fig. 3). This allows identification of clinically relevant contexts (i.e., facing pathologies in the LBD spectrum, other forms of dementia, or having specific clinical scores) in which dopaminergic scintigraphic imaging is efficient.

According to the 2017 DLB consensus criteria, decreased uptake on SPECT is an indicative biomarker that supports the diagnosis of DLB, in addition to the four core clinical features [3]. In our systematic review, we noted that these criteria were respected, and the use of indicative biomarkers for DLB is clearly supported by direct biological biomarkers. There has been no updated consensus criterion since 2017. We identified studies where prodromal DLB could be identified and form a clinical entity, as some studies have shown that screening using SPECT imaging is possible in healthy or paucisymptomatic patients, even years before the diagnosis of DLB [62, 65]. Future research perspectives and biomarker-based research could be anchored towards potential treatment trials in the identified prodromal DLB patients and pave the way for early intervention in pre-dementia syndromes.

## Limitations

We identified several limitations in the various studies we analyzed, such as the heterogeneity of radiotracers that were sometimes used. Furthermore, study designs and outcome measures varied considerably between studies. Extraction of accurate data on true negatives/positives and false negatives/positives was not systematically possible, and a pooled analysis of the studies would most probably entail a large heterogeneity, which is why we decided not to pursue a meta-analysis.

Regarding the technical aspects, image acquisition was usually precisely reported, with details on the injected doses of radiotracers, time-intervals between injection and imaging, types of reconstructions, and algorithms used.

In all the reviewed studies, image analysis was performed either by visual rating alone, semi-quantitative measures using specific binding ratios of the radiotracer in the striatum, or a combination of both methods. However, these methods have the limitation of being user-dependent and lack anatomical standardization. In fact, a few studies in the literature address this issue and point to a promising role of quantitative assessment of DAT loss in the striatum using computer tomography (CT) data acquired on hybrid SPECT/CT equipment [79]. Using CT in order to apply anatomical standardization to dopaminergic scintigraphic imaging, authors like Yokoyama et al. proposed a method that avoids deformation errors due to DaTscan-specific templates lacking structural information [79]. Further research using quantitative assessment is thus required in order to more accurately discriminate Dementia with Lewy bodies and better understand the physiopathology of its distinct clinical features.

## Conclusions

Dopaminergic scintigraphic imaging is an efficient method to diagnose dementia with Lewy bodies and distinguish it from other forms of dementia. This is done through semi-quantitative and visual methods, and very little work has been done including the use of absolute tracer uptake quantification or the CT-guided anatomically standardized methods to accurately measure dopamine transporter decrease in the striatum. Therefore, further research is needed in order to assess dopaminergic degeneration more accurately and to possibly predict the degree of severity and progression of dementia with Lewy bodies.

## Supplementary Information

Below is the link to the electronic supplementary material.

Supplementary file1 (DOCX 76 KB)
