# Supplementary material for: The diagnostic performance of functional dopaminergic scintigraphic imaging in the diagnosis of dementia with Lewy bodies: an updated systematic review
Source: Eur J Nucl Med Mol Imaging. 2023 Mar 15;50(7):1988–2035. doi: 10.1007/s00259-023-06154-y (PMC10199865; doi:10.1007/s00259-023-06154-y)
Supplement: Supplementary file 1 — Supplementary file1 (DOCX 76 KB) [file 259_2023_6154_MOESM1_ESM.docx]

**Supplementary Table 1** Basic study and patient characteristics

| **Authors** | **Year** | **Country** | **Study design** | **Type of patients evaluated** | **No. of patients with DLB** | **Percentage of patients with DLB** | **Age (years)** | **% Male** | **Time since onset of symptoms (years)** | **Mean follow-up (months)** |
| --- | --- | --- | --- | --- | --- | --- | --- | --- | --- | --- |
| Ceravolo et al. [49] | 2003 | Italy | Retrospective single center | Probable DLB and probable AD | 20 | 45 | 70.5±5.3 | 30 | NR | NR |
| Chen et al. [15] | 2021 | USA | Retrospective single center | MCI patients with at least 1 core clinical feature of DLB | 34 | 100 | 68.8 | 88.2 | NR | NR |
| Chiu et al. [60] | 2021 | Taiwan | Retrospective single center | DLB, AD and non-dementia controls | 126 | 45.5 | 76.9±7.6 | 43.7 | NR | 36-48 (3-4 years) |
| Colloby et al. [25] | 2008 | UK | Prospective single center | DLB, AD, elderly controls | 30 | 28.6 | 76±6.5 | 57 | 2.4 ± 1.9 | 3 |
| Colloby et al. [27] | 2005 | UK | Prospective single center | DLB, PD, PDD and HC | 20 | 26 | 74.8±4.0 | 70 | Cognitive symptoms: 2.7±2.1  Parkinsonism: 1.9±1.4 | NR |
| Colloby et al. [28] | 2004 | UK | Retrospective single center | DLB, AD, PD and age-matched HC | 23 | 18 | 75.9±7.1 | 65.2 | 2.46 (SD 1.74)  (29.5m SD 20.9m) | NR |
| Del Sole et al. [61] | 2015 | Italy | Retrospective single center | Probable DLB with a wide range of extrapyramidal signs | 19 | 86 | 78.0±5.5 | 45.5 | NR | 31 (SD 22.3 |
| Donaghy et al. [62] | 2017 | UK | Prospective bi-centric | MCI-LB and MCI-AD | 41 probable MCI-LB | 63 | 75.5±7.6 | 66 | NR | 12 |
| Durcan et al. [63] | 2019 | UK | Prospective bi-centric | MCI-LB and MCI-AD | 48 | 64 | 75.0±8.0 | 64.6 | NR | NR |
| Gupta et al. [9] | 2019 | India | Retrospective single center | Possible DLB and possible PCA | 38 possible DLB patients | 53 | 63.8±8.87 | 65.79 | 2.69 ±1.61 | NR |
| Hansen et al. [64] | 2021 | Germany | Prospective single center | Patients with psychiatric symptomatology and suspected DLB | 67 | 86.6 | 78±0.9 | 55 | NR | 72 (6y) |
| Huber et al. [10] | 2020 | Germany, Switzerland, Australia, Norway, UK | Retrospective multi-centric | DLB patients and HC | 84 | 100 | 72.6±7.0 | 56 | 2.7±2.0 | Mean time interval between ^18^F-FDG-PET and DaT-SPECT = 3.0±4.8 months |
| Iizuka et al. [11] | 2017 | Japan | Retrospective single center | DLB, HC (with normal cognition) | 34 | 100 | 76.9±2.3 | 53 | NR | NR |
| Inagawa et al. [16] | 2020 | Japan | Retrospective single center | DLB and AD | 24 | 100 | 82.4 (73-91( | 58 | NR | NR |
| Iwabuchi et al. [26] | 2022 | Japan | Retrospective single center | DLB, PD and PDD | 24 | 68.6 | 78.5±6.6 | 42 | NR | NR |
| Joling et al. [29] | 2018 | Netherlands | Cross-sectional single center | DLB and PD | 53 | 50 | 67.83±5.94 | 81 | 3.00 (2.00) | NR |
| Joling et al. [30] | 2019 | Netherlands | Cross-sectional single center | Early-stage PD, DLB and HC | 16 | 33.33 | 64.4±4.9 | 50 | 3.00 (2.00) | NR |
| Kamagata et al. [31] | 2017 | Japan | Retrospective observational | DLB, AD/a-MCI and HC | 28 | 24 | 76.1±5.7 | 46.4 | 32.2 m (25.3) | NR |
| Kasanuki et al. [32] | 2017 | Japan | Retrospective single center | Prodromal DLB group (patients with non-motor symptoms associated with DLB, i.e. RBD, olfactory dysfunction and autonomic dysfunction and depression) | 38 | 37.5 | 75.4±6.1 | 44.44 | 72.9±42.9 | NR |
| Kemp et al. [50] | 2011 | UK | Retrospective single center | Suspected DLB patients | 20 | 25 | 79±7.3 | 51 | NR | 12 (DaTscan done 12-24 m months earlier) |
| Kobayashi et al. [17] | 2017 | Japan | Prospective single center | Probable DLB | 34 | 34 | 75±8.3 | 41 | NR | 13 m follow up |
| Lamotte et al. [33] | 2016 | France | Retrospective single center | DLB | 56 | 100 | 74.2±6.9 | 55 | 50.3±30.3 | Patients reviewed from January 2009 to august 2013 |
| Lim et al. [12] | 2009 | Australia | Retrospective single center | Probable DLB and AD | 14 | 58.33 | 72±5.9 | 93 | <3 y | NR |
| Lloyd et al. [51] | 2018 | UK | Retrospective single center | DLB, AD, PD, PDD, FTD and HC | 12 | 26 | 75.6±6.3 | 58 | NR | NR |
| Maltais et al. [52] | 2020 | USA | Retrospective single center | Probable DLB, AD demented, mixed AD/probable DLB, PD with MCI, CBS, iRB and behavioral FTD | 12 | 50 | 80.5±9.7 | 76 | NR | NR |
| McKeith et al. [68] | 2007 | 40 European sites | Phase III multicentric prospective study | Probable DLB, possible DLB and non-DLB dementia | 94 probable DLB, 57 possible DLB | 29 | 74.2±6.6 | 63 | NR | Follow up from November 2003 and August 2005 |
| Miyagawa et al. [13] | 2021 | USA | Retrospective single center | DLB and ADem | 35 | 28.6 | 67.3±9.7 | 94 | 5.9 (4.9) | NR |
| Miyamoto et al. [58] | 2020 | Japan | Retrospective single center | iRBD and LBD | 6 | 24 | 70.0±6.2 | 72 | NR | 5 year follow up |
| Morgan et al. [34] | 2012 | UK, check if multicentric with Walker | Prospective single center (same as Walker 2002 and Walker 2007) | DLB, AD and FTD | 10 | 34.5 | 77.9±8.4 | 60 | 3 years | NR |
| Nakahara et al. [18] | 2021 | Japan | Retrospective single center | LBD: DLB, PD and PDD | 10 | 11.9 | 74.7±9.3 | 38 | 1.9 (2.0) | NR |
| Nicastro et al. [35] | 2017 | Switzerland | Retrospective single center | DLB and AD | 93 | 83.8 | 76.9±6.8 | 63 | 1.78±1.66 | NR |
| Nicastro et al. [53] | 2018 | Switzerland | Prospective observational study, single center | DLB, AD, FTD, CJD, VD, mixed AD-VD, PD, MSA, CBS, PSP, VP, NPH and HC | 53 | 59.6 | NR for DLB (71.7±11.6 for all subjects) | NR (52% males for all subjects) | NR | Scans collected between 11^th^ September 2013 to 4^th^ October 2016 |
| Nicastro et al. [14] | 2021 | Switzerland | Retrospective single center | DLB | 34 | 100 | 72.5±6.8 | 62 | 1.35±1.1 | NR |
| O’Brien et al. [36] | 2004 | UK | Prospective cohort study | DLB, AD, PD, PDD and HC | 23 | 14 | 75.9±7.1 | 65 | 29.5 (20.9), months | NR |
| O’Brien et al. [37] | 2009 | UK (patient over 40 European sites) | Prospective multicentric (same cohort as McKeith et al., see below) | Probable DLB, possible DLB and Non-DLB dementia | 68 probable DLB, 44 possible DLB | 29 | 73.7 | 62 (probable DLB) | Showed cognitive decline over 12 months | 12 |
| Oliveira et al. [59] | 2021 | Portugal | Retrospective single center | DLB, AD, PD, HC and Other diagnoses (FTD, VD, CBD) | 8 | 15 | 76±10 | 50 | NR | NR |
| Pilotto et al. [38] | 2019 | Italy | Prospective single center | DLB, PD and HC | 41 | 27 | 72.4±6.0 | 73 | 2.1±1.3 | 2 to 4 years follow up |
| Ransmayr et al. [39] | 2001 | Austria | Cross-sectional single center | DLB, PD and HC | 20 | 37 | 71.1±6 | 65 | 5.1±2.8 | NR |
| Roberts et al. [19] | 2021 | UK | Prospective cohort study single center | MCI-AD, probable MCI-LB, possible MCI-LB and HC | 42 probable MCI-LB, 17 possible MCI-LB | NR | 74.8±6.1 (probable MCI LB) | 95 | NR | 1.6±0.85 |
| Roberts et al. [54] | 2021 | UK | Prospective cohort study, single center | MCI-AD/AD, probable MCI-LB/DLB and possible MCI-LB/DLB | 61 = Total probable DLB/MCI-LB : 40 (abnormal DaTscan) + 21 (normal DaTscan) | 42 | 74.6±7.1 | 79 | NR | 2.0 (0 to 7 years in study) |
| Roselli et al. [40] | 2009 | Italy | Prospective single center | DLB | 18 | 100 | 76.4±3.6 | 61 | NR | NR |
| Sakamoto et al. [20] | 2020 | Japan | Retrospective single center | Possible DLB (117) | 47 | 40 | 71.0±11.5 | 68 | NR | NR |
| Shimizu et al. [21] | 2016 | Japan | Retrospective single center | DLB and AD | 76 | 57 | 80.2±4.9 | 55 | 3.2 y ±0.9 y | March to september 2014 |
| Shimizu et al. [22] | 2017 | Japan | Retrospective single center | DLB and AD | 32 probable DLB | 50 | 80.3±4.0 | 50 | 2.8 y ±0.9 y | 2 months (to do all the imaging) |
| Siepel et al. [65] | 2013 | Norway | Longitudinal case/cohort study over 2-5 years; single center | Patients fulfilling clinical DLB criteria with a negative (123I)FP-CIT SPECT scan (S−CF+) and patients with an abnormal scan not fulfilling clinical DLB criteria (S+CF−) | S-CF+ : n=3, S+CF- : n=7 | 70 | 71±9.0 | 57 | NR | Observation time = 3.4 y (2-5) |
| Siepel et al. [41] | 2016 | Norway | Longitudinal cohort study (DemWest cohort) | Probable DLB | 35 | 10 | 74.3±7.4 | 54 | 3.4 y ±2.0 | Average time between initial clinical evaluation and date of FP-CIT = 6.5 m |
| Spehl et al. [42] | 2015 | Germany | Retrospective single center | DLB, FTD and AD | 12 | 35 | 71±5 | 50 | 24m±17m | NR |
| Taylor et al. [43] | 2007 | UK | Cross-sectional single center | DLB, AD and PDD | 28 | 28 | 76.9±6.7 | 60.7 | 30.6±25.5 m | NR |
| Thomas et al. [55] | 2019 | UK | Prospective cohort study, single center | Probable MCI-LB, possible MCI-LB and MCI-AD | 33 probable MCI-LB and 15 possible MCI-LB | 64 (possible and probable MCI-LB) | 75.0±7.5 (if probable MCI-LB) | 66.66 | NR | 1.5 y (mean) |
| Tiraboschi et al. [23] | 2016 | Italy | Prospective longitudinal study | DLB, AD and FTD | 30 | 51 | 73.5±4.8 | 70 | NR | Recruited between 01.07.12 and 31.12.2012 |
| Treglia et al. [24] | 2012 | Italy | Prospective single center | DLB, PD, MSA, CBD, PSP, VP, AD, FTD and VD | 20 | 20 | 72.5±10 | 60 | NR | NR |
| Van de Beek et al. [44] | 2021 | Netherlands | Longitudinal prospective cohort study, single center | DLB | 100 | 100 | 69±6 | 90 | 4±3 | NR |
| Van der Zande et al. [45] | 2016 | Netherlands | Case-control study | Probable DLB | 18 | 100 | 72.5 (56.5-82) | 77 | 3.5 (1-8) | 24 (8-36 m) |
| Van der Zande et al. [46] | 2020 | Netherlands | Prospective single center | Probable DLB with and without concomitant AD pathology | 52 | 100 | 67.85 | 86.5 | NR | NR |
| Walker et al. [66] | 2002 | UK | Prospective single center | DLB, AD, PD and HC | 27 | 34 | 77.3 (7.9) | 37 | NR | NR |
| Walker et al. [47] | 2004 | UK | Prospective single center | DLB, AD, PD and HC | 21 | 37.5 | 78.6 (7.6) (at time of scan) | 24 | 3.9 (2.2) | NR |
| Walker et al. [56] | 2007 | UK | Prospective single center | DLB, AD (clinical diagnosis) | 8 | 40 | 76.5 (9) (at time of scan) | 37.5 | NR | 10 y |
| Walker et al. [57] | 2015 | 6 European countries | Randomized controlled, open label multicentric (21 centers) | Possible DLB | 170 possible DLB | 100 | 75 (7.25) | 54.7 | NR | 6 m |
| Ziebell et al. [48] | 2013 | Denmark | Prospective single center | DLB | 51 | 100 | 74.5±8.1 | 60 | Cognitive symptoms: 65.4 ± 17  Parkinson core motor symptoms: 25.1±19.7 | 16 m |

NR not reported, DLB Dementia with Lewy bodies, MCI Mild cognitive impairment, AD Alzheimer’s disease, ADem Alzheimer’s disease dementia, PD Parkinson’s disease, PDD Parkinson’s disease with dementia, FTD Frontotemporal dementia, CBS Corticobasal syndrome, PSP progressive supranuclear palsy, MSA multi-system atrophy, CJD Creutzfeldt-Jakob Disease, VD vascular dementia, VP vascular parkinsonism, NPH normal pressure hydrocephalus, iRBD idiopathic rapid eye movement sleep behaviour disorder. Probable MCI-LB >= 2 diagnostic features, Possible MCI-LB 1 Diagnostic feature, PCA posterior cortical atrophy (Bensons syndrome, a variant of AD), a-MCI anamnestic (amnestic?) mild cognitive impairment, HC Healthy controls.
